# Programmable Continuous Electrowetting of Liquid Metal for Reconfigurable Electronics

**DOI:** 10.1002/adma.202506383

**Published:** 2025-09-15

**Authors:** Wedyan Babatain, Christine Park, Deiaa M. Harraz, Ozgun Kilic Afsar, Cedric Honnet, Sarah Lov, Jean‐Baptiste Labrune, Michael D. Dickey, Hiroshi Ishii

**Affiliations:** ^1^ Media Lab Massachusetts Institute of Technology Cambridge MA 02139 USA; ^2^ Department of Chemistry Massachusetts Institute of Technology Cambridge MA 02139 USA; ^3^ Department of Chemical and Biomolecular Engineering North Carolina State University (NCSU) Raleigh NC 27695 USA

**Keywords:** electrochemical actuation, electrowetting, fluidic valves, liquid metal, programmable matter, reconfigurable electronics

## Abstract

Dynamic manipulation of the shape and position of liquid metal (LM), a conductive and deformable conductor, presents new opportunities for reconfigurable electronics, fluidic logic, and soft‐actuation systems. This study combines continuous electrowetting (CEW) with electrochemical modulation of the interface of LM in electrolyte to achieve tunable and directional LM manipulation in 2D spaces. A key finding is that under a fixed external electric field, the LM moves in a direction that depends on its electrochemical potential. The LM potential is controlled using a substrate featuring patterns of laser‐induced graphene (LIG) since it is non‐wetting to LM and electrically conductive. This strategy enables a range of functionalities, including “valves” for on‐demand LM control, LM droplet sorting, feedback sensing, and fluidic logic gates. The strategy can also control the motion of LM droplets across 2D spaces. Finally, it is utilized within a reconfigurable circuit platform where the LM functions as a dynamic interconnect for sequential activation, parallel switching, and self‐healing circuits. By coupling the electrically‐driven motion of LM and the versatility of LIG patterning, this work establishes a versatile framework for reconfigurable electronics, programmable fluidic systems, and adaptive systems.

## Graphical abstract

This work introduces a programmable continuous electrowetting (CEW) approach for the dynamic manipulation of liquid metal (LM), enabling directional control via electrochemical modulation. By leveraging laser‐induced graphene (LIG) substrates, a wide range of utilities is demonstrated, including fluidic “valves,” logic gates, and reconfigurable circuits. This approach contributes to the development of next‐generation reconfigurable electronics and digital fluidic systems.

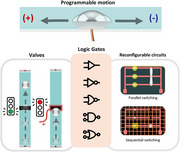

## 1 Introduction

Liquid metals (LMs), particularly gallium‐based alloys, exhibit unique properties, including high electrical and thermal conductivity, fluidity, and responsiveness to external stimuli (electric field, magnetic field, acoustic field, chemical field, temperature, light).^[^ ^1^, ^2^, ^3^ ^]^ These characteristics, combined with their electrochemically tunable interfacial tension,^[^ ^4^, ^5^, ^6^ ^]^ make liquid metals a promising class of material for applications in soft robotics,^[^ ^7^, ^8^, ^9^, ^10^ ^]^ reconfigurable electronics,^[^ ^11^, ^12^, ^13^ ^]^ actuators,^[^ ^14^, ^15^, ^16^ ^]^ sensors,^[^ ^17^, ^18^, ^19^, ^20^ ^]^ and microfluidics.^[^ ^21^ ^]^ This paper focuses on controlling the motion of liquid metal droplets (LMDs). Achieving precise control of the position and motion of LMDs expands their application space^[^ ^22^ ^]^ to include drug delivery,^[^ ^23^, ^24^ ^]^ micro‐object transportation,^[^ ^25^, ^26^ ^]^ fluid pumping,^[^ ^27^, ^28^ ^]^ robotic actuation,^[^ ^29^, ^30^ ^]^ artificial muscles,^[^ ^29^ ^]^ and linear actuators.^[^ ^31^ ^]^ Existing methods for actuating liquid LMDs utilize magnetic,^[^ ^32^ ^]^ optical,^[^ ^11^, ^33^ ^]^ chemical,^[^ ^34^, ^35^ ^]^ or acoustic^[^ ^36^ ^]^ forces.

In this paper, we use continuous electrowetting (CEW) to move and manipulate LMDs.^[^ ^37^ ^]^ CEW involves placing the LMD in an electrolyte solution. A direct current (DC) voltage applied between two electrodes establishes an electric field within the solution. Throughout this paper, we refer to these two electrodes as the anode (+) and cathode (−). The field shifts the electrical double layer (charge) that forms at the interface between the LMD and electrolyte. The shift results in an interfacial tension gradient that generates a Marangoni force that causes the droplet to move.^[^ ^5^, ^6^ ^]^ Interestingly, the slope of this surface tension gradient changes as a function of the potential regime of the LMD; the behavior under reducing potentials differs from that under oxidizing potentials. At reducing potential regimes, the surface tension increases with increasing potential; however, under oxidizing potential regimes, a surface oxide forms, causing the surface tension to decrease with increasing potential.^[^ ^4^ ^]^ A NaOH solution is often used as the electrolyte to keep the metal free of native oxide, which would otherwise slow its movement.^[^ ^38^, ^39^ ^]^ In alkaline electrolytes (such as NaOH solution), gallium liquid metal naturally forms a negative charge on its surface, causing it to move toward the anode under an externally applied field. We reasoned that since the direction of movement depends on the polarity of the charge (+/−) on the surface of the LMD, we could cause the LMD to move toward the cathode by setting a more positive potential at the LM‐solution interface, essentially modulating its CEW behavior on demand.

To control the potential of the LMD, we patterned laser‐induced graphene (LIG)^[^ ^40^ ^]^ on the substrate since LIG is non‐wetting (toward LM), conductive, and electrochemically active. When LMD encounters regions of the substrate patterned with LIG, its direction of motion reverses, even under the same applied external field, now migrating toward the cathode due to electrochemical oxidation.^[^ ^41^ ^]^ This reversal arises due to a shift in the LM surface potential upon contacting. This response can be further modulated by applying an external potential to the LIG itself. Thus, the movement and direction of LMD can be programmed by controlling the potential of LIG and its pattern, enabling a variety of programmable functionalities. In addition to controlling the charge on the surface, the oxidation of the liquid metal improves its adhesion with the LIG substrate, therefore making the metal easier to manipulate (otherwise, LMDs readily “slip” across a surface in response to small, undesirable disturbances). The approach here overcomes some limitations of existing CEW manipulation platforms, including geometric constraints, limited LMD directional control, and electrode/substrate incompatibility. Herein, we demonstrate the versatility of the approach through practical applications, including surface‐enabled dynamic fluidic “valves,” programmable logic gates for digital operations, and two‐dimensional (2D) platforms for discrete LMD locomotion and reconfigurable circuits.

## 2 Results and Discussion

### 2.1 Modulating Liquid Metal Dynamics within Continuous Electrowetting Fields

**Figure 1:**
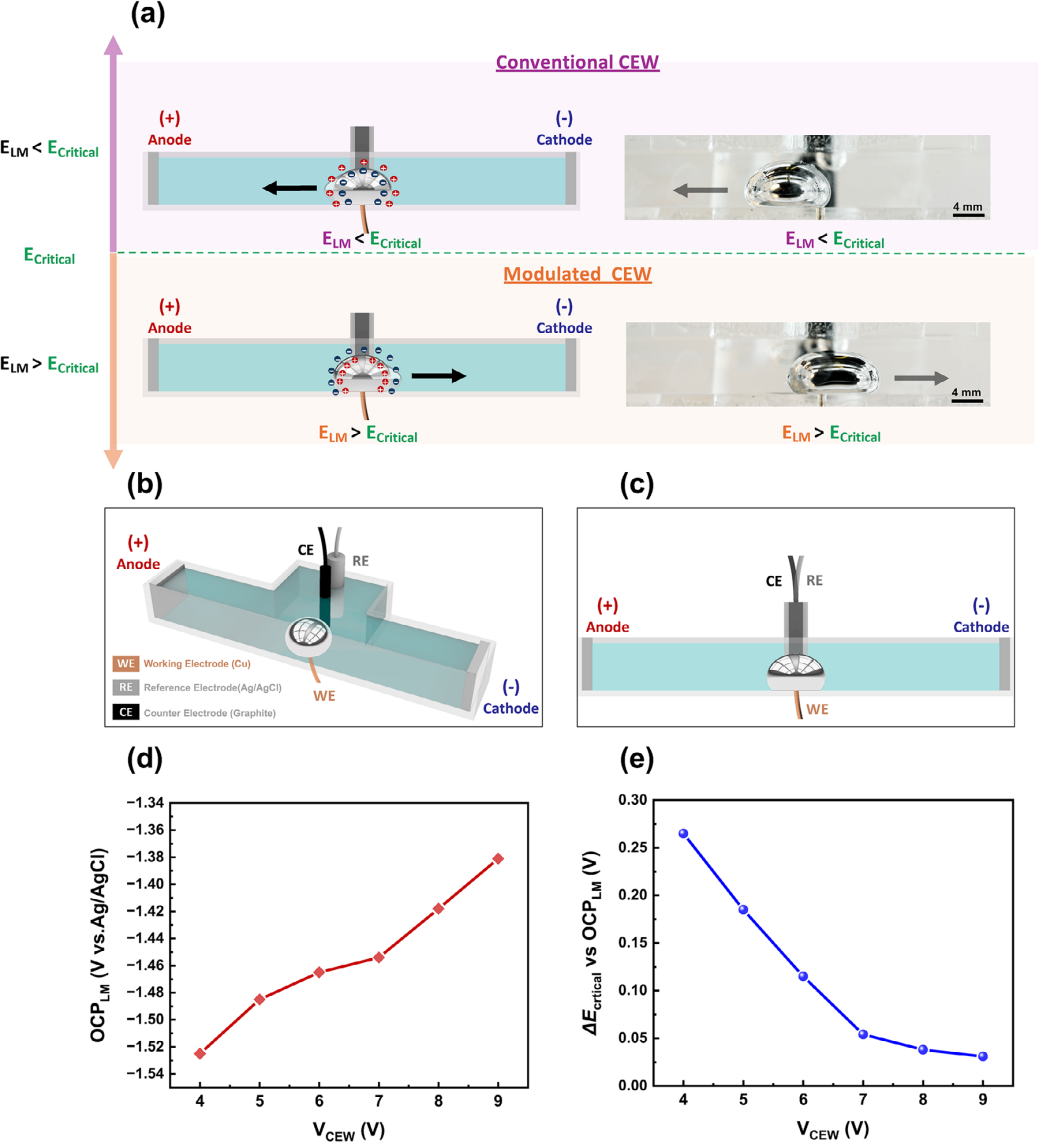
Experimental setup and characterization of programmable liquid metal continuous electrowetting (CEW). a) Schematic illustration comparing conventional LM CEW (top) and modulated LM CEW (bottom). In conventional CEW, LMD moves toward the anode when the LM potential (E_LM)_ is below a critical threshold (E_critical_). In modulated CEW, a localized potential applied to the LM raises E_LM_ above E_critical_, reversing the direction of the motion. Corresponding side‐view photographs show LM directional movement for each case. b) Isometric view and c) side view of the experimental setup, showing the working electrode (WE) wetted to LM, counter electrode (CE), and reference electrode (RE) within the channel. d) Open circuit potential (OCP) of the LM as a function of the applied CEW voltage (V_CEW_). e) Change in critical potential (ΔE_critical_) for LM motion relative to OCP under varying applied V_CEW_.

Our approach enables directional switching, allowing LMDs to move toward either the external anode or the cathode depending on the applied potential to the LMD. In a conventional CEW setup in NaOH, LMD has a negatively charged surface that attracts cations (sodium ions *Na* ^+^) from the solution,^[^ ^27^ ^]^ forming an electrical double layer (EDL) that is shown in **Figure** **1**a

Consequently, during CEW, LMD moves toward the anode as shown in the top panel of Figure 1a.^[^ ^42^ ^]^ Further mechanism details are found in Note S3 (Supporting Information).

Applying a sufficient anodic (+) potential directly to the liquid metal E_LM_ via a working electrode (WE), we find that we can reverse the movement toward the cathode as depicted on the bottom panel of Figure 1a. This change in direction can be seen in Video S1 (Supporting Information) with the experimental setup shown in Figure S1 (Supporting Information). The potential at which the LMD changes direction is called E_critical_, which relates to the charge on the surface of the LMD. The approach consists of two circuits. Circuit 1 consists of two graphite rod electrodes at the two ends of the channel, as depicted in the schematics of Figure 1b,c. V_CEW_ is the applied voltage across these rods that establishes the field necessary for CEW. Circuit 2 applies a potential to the LMD to modulate its surface. Here, a copper wire working electrode is brought into contact with the LMD, a graphite rod counter electrode, and asilver/silver chloride (Ag/AgCl) reference electrode adjacent to the LMD, as illustrated in Figure 1b.

We sought to quantify E_critical_ and determine if there is any dependence of E_critical_ on the potential V_CEW_ applied to circuit 1. First, the open circuit potential (OCP) of the LMD in 1 M NaOH was measured as a function of external CEW voltage, without any potential applied to the LMD. Figure 1d shows that as the CEW voltage increases, the OCP of LMD shifts slightly more positively. Next, a series of chronoamperometry (CA) experiments was performed to set the potential of the LMD. The set potentials were stepped to more anodic potentials until the locomotion of the LMD reversed direction under a given V_CEW_. ΔE_critica_ is the difference between the critical potential at which the LMD reverses direction and the OCP measured at a given V_CEW_. Figure 1e shows that ΔE_critical_ decreases slightly as V_CEW_ increases, indicating that a stronger CEW field lowers the required potential for LMD motion reversal, making it more susceptible to motion reversal. Additionally, we measured the current in circuit 1 (i.e., through the LM) and found that the change in direction corresponds to a switch from negative to positive current, indicating oxidation (Figure S2, Supporting Information). The oxidation of the metal is apparent based on its increased adhesion to the substrate relative to oxide‐free conditions of LM. In these experiments, LMD adhered to the Cu working electrode, limiting its motion. The next section explores LIG electrodes that allow the LMD to move more freely.

### 2.2 Liquid Metal Manipulation Enabled by Laser‐Induced Graphene Substrates

To control the potential applied to LM (and thus, the CEW directional response), it is necessary to make electrical contact with LM. LM can alloy with metallic substrates (e.g., copper), leading to undesirable interactions such as irreversible sticking, motion constraints, and long‐term electrode degradation.^[^ ^43^, ^44^ ^]^ While Figure 1 demonstrated directional control using direct Cu wire contact, practical applications require a more versatile approach that can provide both passive modulation (through surface contact) and active control (through applied potentials).To overcome this limitation, we used LIG as a compatible material for LM manipulation^[^ ^20^, ^45^, ^46^ ^]^ (Video S2, Supporting Information). Because LIG is electrochemically active when it contacts LMD, it can passively change the CEW behavior of LMD, causing it to move toward the cathode. Yet, because LIG is conductive, it can also be used actively to control the potential applied to the LMD, manipulating its response to CEW fields. Additionally, LIG is easily patterned into polyimide films using accessible laser tools, allowing its integration into one‐dimensional (1D) channels and 2D platforms. Notably, the pattern of LIG directly influences the CEW behavior of LMD, introducing an additional layer of versatile control.

#### 2.2.1 Passive Substrate

**Figure 2:**
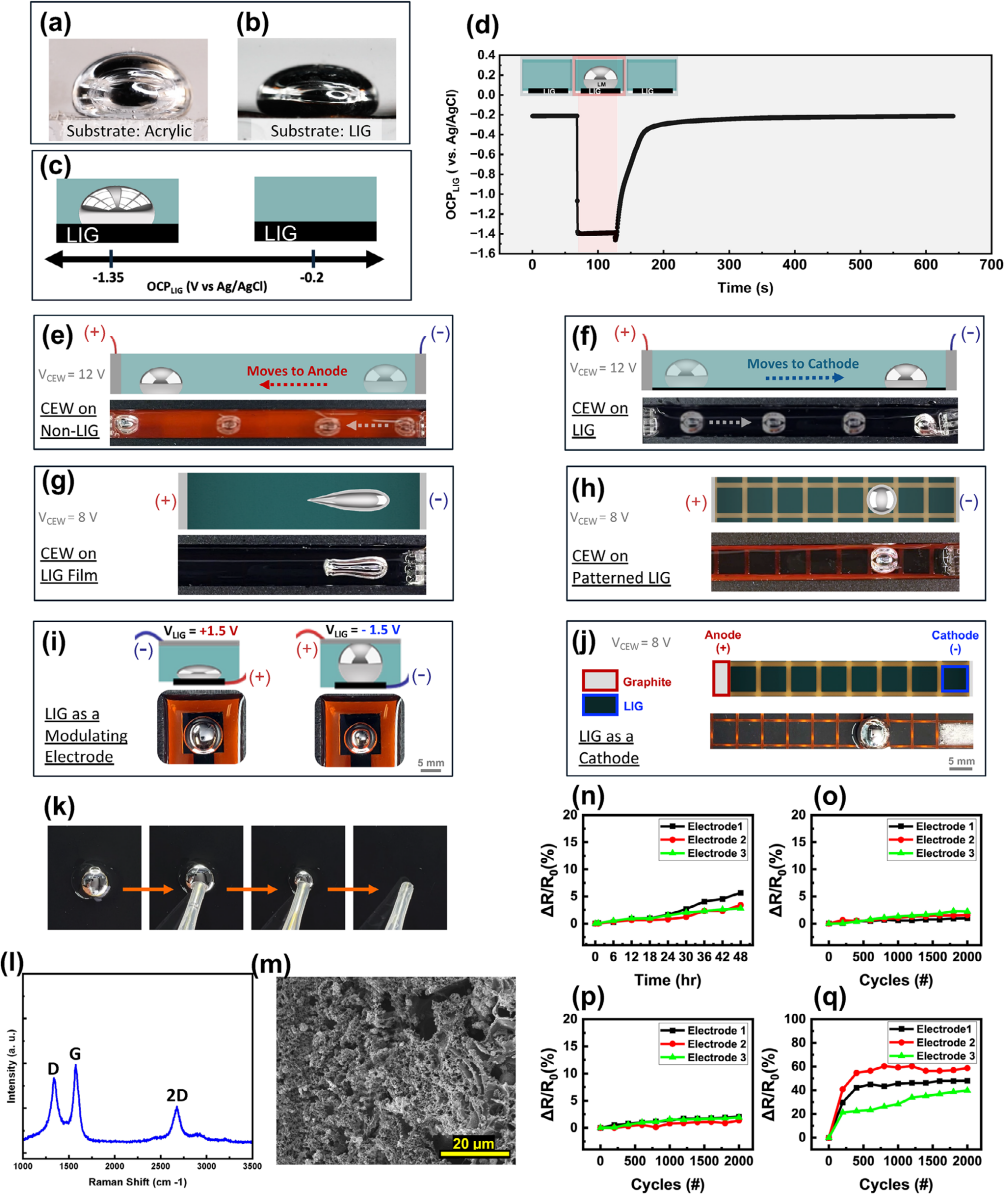
Liquid metal manipulation enabled by laser‐induced graphene substrates. a,b) Side view of liquid metal (LM) droplet on (a) non‐LIG (acrylic) and (b) LIG surfaces, showing droplet shape differences due to surface interaction. c) Static open circuit potential (OCP) of LIG in 1M NaOH bath, shifting from −0.2 V (bare LIG electrode) to −1.35 V upon LM contact with the LIG electrode. d) Dynamic OCP of LIG electrode over time, showing potential shifts as LM contacts the electrode surface and its recovery as it detaches from the surface. e,f) LM motion under CEW on (e) non‐LIG polyimide substrate and (f) LIG substrate, showing reversed directionality. g,h) LM shape during CEW on (g) LIG film, where it elongates as it moves, and (h) grid‐patterned LIG, where it retains a discrete droplet shape. i,j) LIG as an active modulating electrode, applying (i) oxidative potential (+1.5 V) to lower the surface tension of the LM, causing it to spread, and (j) reductive potential (−1.5 V) to increase the surface tension, causing it to bead up. k) Deposition and retraction of LM on LIG substrate, showing no visible residue left behind and non‐wetting interaction. l) Raman spectrum confirming the characteristic D, G, and 2D peaks of LIG. m) SEM image showing the porous, fibrous morphology of LIG. n) Long‐term stability of LIG electrodes in 1 M NaOH, showing relative resistance change (ΔR/R0) of the LIG electrode over 48 h.o) Relative resistance change of LIG electrodes used as modulating electrodes applying +0.5 V oxidative potential for 2000 cycles. p) Relative resistance change of LIG electrodes applying −0.5 V reductive potential for 2000 cycles.q) Relative resistance change of LIG electrodes used as cathodes applying −10 V for 2000 cycles during CEW operation.

**Figure** **2** demonstrates how LIG passively modulates LM surface properties. Figure 2a,b presents side images, showing the shape of a LMD on LIG compared to a non‐LIG substrate. On non‐LIG substrates (polyimide/acrylic), LMD retains a spherical droplet shape (Figure 2a). However, on LIG it flattens (Figure 2b), indicating a decrease in interfacial tension due to electrochemical oxidation.^[^ ^41^, ^47^, ^48^ ^]^ Electrochemical oxidation is known to lower the interfacial tension of LM.^[^ ^4^ ^]^ In addition to changing the surface chemistry, the formation of the oxide makes the LMD more adhesive to the substrate,^[^ ^7^ ^]^ thereby mitigating the challenge of high surface tension that makes LMDs difficult to control (that is, it can easily roll away or move in response to vibrational disturbances). It is important to note that this oxidation‐mediated adhesion is different from the strong, irreversible adhesion observed between LM and metallic substrates such as copper.^[^ ^48^ ^]^ In the case of copper, gallium‐based LM forms intermetallic bonds through alloying, leading to wetting that severely restricts detachment and limits locomotion. In contrast, the adhesion observed on LIG arises from electrochemical oxidation at the interface, which produces a reversible, voltage‐tunable effect. This adhesion is desirable and forms the basis of many programmable behaviors that will be introduced in our system.

To confirm and quantify the electrochemical activity of LIG, we measured the OCP of a LIG electrode. As shown in Figure 2c, the OCP of LIG in a 1 M NaOH is −0.2 V, and the OCP of LM in 1 m NaOH is −1.53 V (all voltages are reported relative to Ag/AgCl unless otherwise noted). Yet, when LM is brought into contact with LIG, the OCP of the combined LIG‐LM electrode is −1.35 V, which is more oxidative than that LM alone. The oxidation of LM is expected, given the decrease in LM droplet surface tension. Recent studies have shown that oxidation begins to occur at −1.4 V.^[^ ^49^ ^]^ Dynamic OCP measurements shown in Figure 2d further demonstrate this electrochemical effect and its reversibility in real‐time. When LMD touches LIG, the LIG OCP shifts to −1.35 V and restores to its original value upon removal of the LMD.

This interaction impacts the behavior of LMD under CEW. As mentioned before, LMD moves toward the anode on polyimide surfaces under CEW voltage (Figure 2e). However, Figure 2f shows that LMD reverses its motion and moves toward the cathode when on a LIG‐patterned polyimide substrate (Video S3, Supporting Information).

As demonstrated in Figure 2g,h, the pattern of LIG influences the dynamics of LM locomotion (Video S4, Supporting Information). In a film of LIG, LM elongates during locomotion due to continuous oxidation, which lowers surface tension and promotes adhesion. On grid‐patterned LIG, LMD maintains its discrete droplet shape. The gaps between LIG patterns were optimized to allow LMD to momentarily regain higher surface tension, maintaining its discrete droplet form. This behavior is advantageous for applications requiring discrete control of the droplet (Note S10, Supporting Information).

#### 2.2.2 Active Substrate

Beyond serving as a passive substrate, LIG can also function as an active substrate for manipulating LMDs. This active role is achieved by applying a voltage between the LIG electrode (V_LIG_) and a counter electrode present in the solution to modulate the interfacial tension of LM. When the LMD touches the LIG, it experiences the potential V_LIG_. When a reductive potential is applied through the LIG, the LMD beads up due to the reduction of the surface oxide layer. On the other hand, when an oxidative potential is applied, the LMD flattens as the oxide layer forms. This is shown in Figure 2i, where the cross‐sectional view schematic illustrates this effect while the top view images show the increase or decrease in the LMD diameter, reflecting the modulation of its interfacial tension upon potential application.

LIG can also be used to replace the graphite cathode (in Figure 1) used to apply the field for CEW. Figure 2j presents the schematic configuration, and the corresponding experimental images demonstrate its operation, including visible hydrogen bubbling at the cathode due to electrolysis. While LIG remains stable as a cathode with high applied potentials (up to 10 V), using it as an anode at such a high potential leads to degradation due to electrochemical oxidation. The anodic reaction induces oxygen evolution and carbon oxidation, which damages the LIG structure, causing delamination of the electrode. Thus, we have only implemented LIG as a cathode for such high potentials.

Figure 2k shows the compatibility of LIG with LM; a pipette can remove the LMD from the LIG without leaving residue or adhesion. Raman spectra (Figure 2l) confirm the presence of LIG, and the SEM image (Figure 2m) shows its porous nature. To confirm the stability of LIG as an electrode, several experiments were performed. The long‐term stability of the LIG electrode was measured by monitoring the relative change of resistance of the LIG for 48 h when immersed in 1M NaOH solution, showing minimal degradation over time (Figure 2n). Cyclic stability tests were conducted by applying V_LIG_ = ±0.5 V (modulating electrode mode) and V_LIG_ = −10 V (cathode mode) for 2000 cycles, demonstrating minimal resistance increase of the electrodes (Figure 2o–q). Notably, in Figure 2q, applying −10 V as a cathode initially leads to a ∼50% increase in relative resistance, likely due to water electrolysis. However, after the first 100 cycles, the resistance stabilizes, indicating that while an initial baseline increase occurs, LIG remains functionally stable over extended actuation cycles afterward. To further assess the mechanical durability of LIG under long‐term dynamic contact with moving LMDs, we conducted a dedicated mechanical cycling test simulating continuous LMD motion for 10000 cycles. Results confirmed both electrical and physical stability of the LIG electrodes over time. Full details and characterization are provided in Supporting Information.

### 2.3 Practical Applications

#### 2.3.1 Programmable LMD LIG Valves

**Figure 3:**
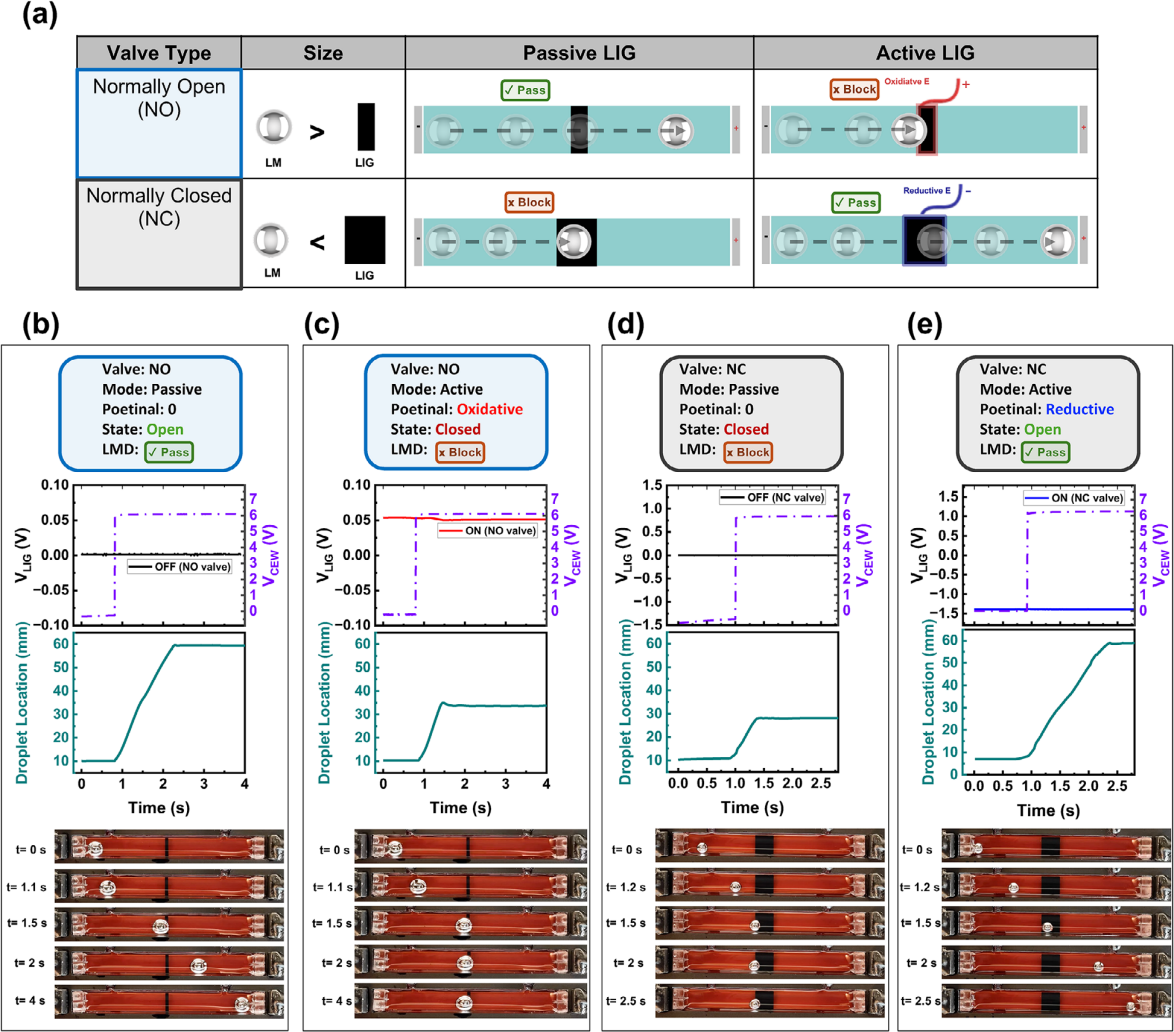
Practical applications of LIG valves for programmable LMD control. a) Classification of LIG‐based valves, showing normally open (NO) and normally closed (NC) configurations in both passive and active modes for size‐dependent LMD control. b,c) NO valve functionality: (b) Passive mode allows the LMD to pass freely, while (c) Active mode applies an oxidative potential to close the valve. d,e) NC valve functionality: (d) Passive mode blocks the LMD, while (e) active mode applies a reductive potential to open the valve. Plots show valve potential (V_LIG_), CEW voltage (V_CEW_), and LMD position over time, with sequential snapshots illustrating LMD movement.

Building on the demonstrated ability of LIG to modulate LMD behavior, we now showcase some of its practical applications. One intriguing utilization is creating LIG surface‐enabled “valves” for LMD control within fluidic channels. Here, a “valve” describes the surface that will either allow or block LMD movement under CEW. **Figure** **3**a presents a classification of these valves, their design, and operational modes. LIG's intrinsic oxidation effect on LM enables valves that consist of LIG strips. By adjusting the LMD diameter relative to the width of the LIG strip, we created two valve types: normally open (NO) and normally closed (NC), summarized in Figure 3a. The NO valve is formed when the diameter of the LMD is larger than the width of the LIG strip. Passively, this valve remains open because the small LIG surface area is insufficient to immobilize the LMD. This valve can be actively closed by applying a small oxidative potential to the LIG electrode, altering LMD's surface, and effectively blocking its locomotion across the channel. In contrast, the NC valve is formed when the diameter of the LMD is smaller than the width of the LIG. Passively, this valve remains closed as the larger LIG contact area is sufficient to prevent the LMD from advancing through the channel under the CEW effect. This NC valve can be actively opened by applying a small reductive potential through the LIG, reducing LMD's surface and allowing it to pass through the valve. These valves operate solely via surface electrochemical modulation, requiring no mechanical moving parts or additional components.

##### Size‐Dependent Control of Passive LIG Valves

The operation of passive and active LIG valves is size‐dependent, governed by the ratio of LMD diameter to LIG width and the CEW voltage magnitude. To study this effect, we defined a size ratio metric, relating the LMD diameter to the LIG width.

(1) $$\mathrm{Size}\;\mathrm{ratio}=\frac{\mathrm{LMD}\;\mathrm{diameter}\left(\mathrm{mm}\right)}{\mathrm{LIG}\;\mathrm{width}\left(\mathrm{mm}\right)}$$

Using this metric, we tested two NO valve widths (1 and 3 mm) and two NC valve widths (6 and 8 mm) across varying LMD diameters and CEW voltages (Tables S2 and S3, Supporting Information). For a 1 mm‐wide NO valve, a size ratio of 5.5 covered its operational range across 4–8 V of CEW voltage. Similarly, for a 3 mm‐wide NO valve, a size ratio of 2 ensured its operation under the same CEW range. For NC valves, a size ratio of 1.25 was sufficient for reliable operation in both 6 and 8 mm‐wide NC valves for the same CEW range. Other factors, such as electrode spatial arrangement and electrolyte concentration, would affect these operational ranges. Figure 3b–e demonstrate the valves' functionality, showing their type (NO or NC), mode (passive or active), time‐series plots of the voltage applied to the valve (V_LIG_), the applied CEW voltage (V_CEW_), the location of the LMD over time, and snapshots of the experiments. Figure 3b shows a passive NO valve (1mm), LMD passes freely as V_LIG_ = 0. In contrast, Figure 3c demonstrates the active mode of the same valve, applying V_LIG_ = +50 mV blocks LMD movement. Figure 3d shows a passive NC valve (6mm); LMD is blocked even when V_LIG_ = 0. Finally, in Figure 3e, applying a reductive potential of V_LIG_ = −1.4 V actively opens the valve, allowing the LMD to pass. (Video S5, Supporting Information).

##### LMD Sorting via LIG Valves

**Figure 4:**
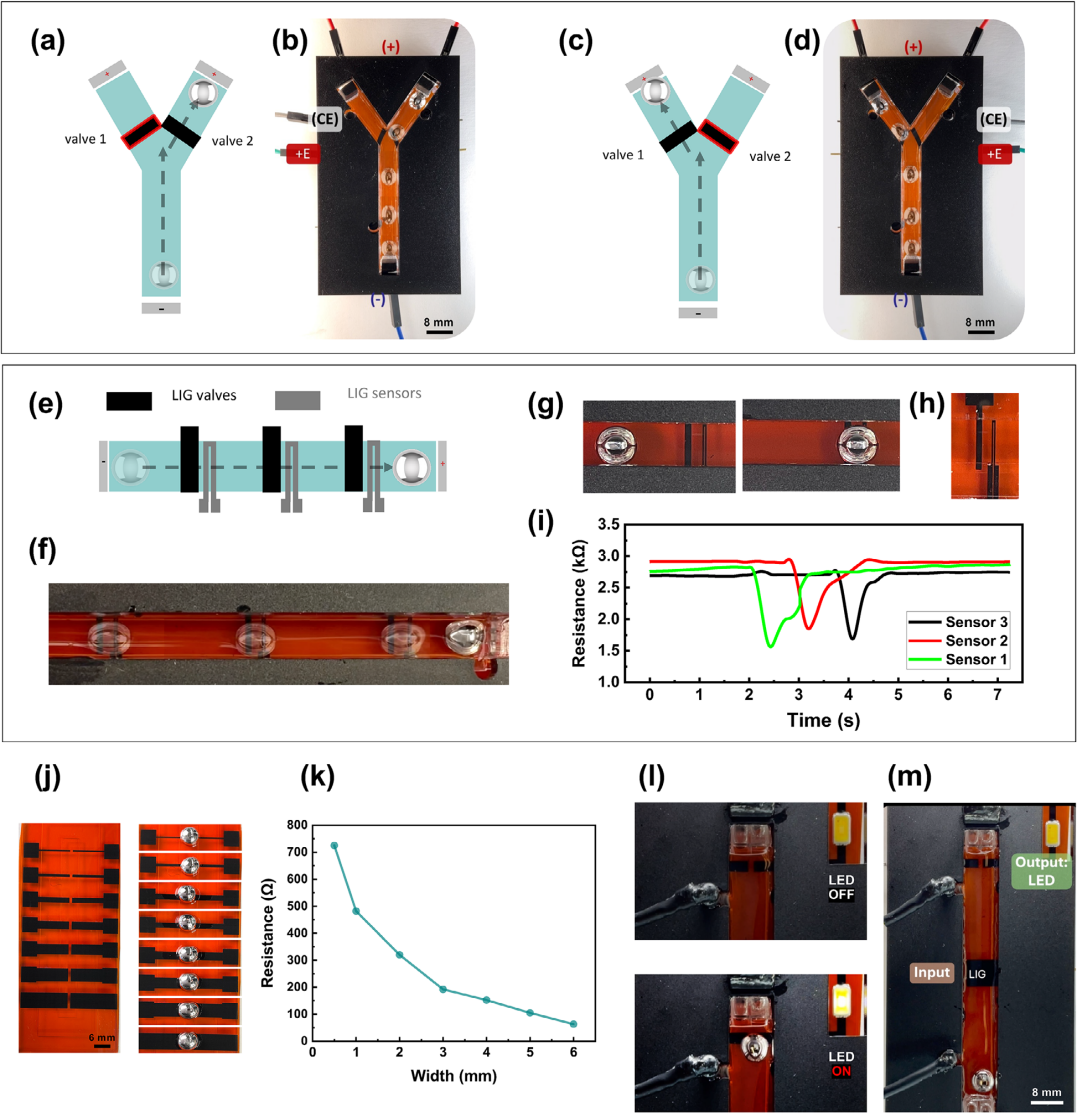
Practical applications. a–d) Y‐shaped channel for liquid metal droplet (LMD) sorting using LIG‐based valves. a,c) Schematics showing valve operation, where applying an oxidative potential blocks LMD entry into a branch. b,d) Experimental demonstration of selective path control. e–i) LIG resistive sensors for real‐time LMD detection. (e) Schematic of LIG sensors placed after each valve. (f) Experimental images showing LMD progression through the channel and sensor array. g,h) Close‐up views of LMD contacting a sensor. i) Resistance measurements tracking sequential sensor activation as the LMD passes, confirming position detection. j–k) LIG as an electrical interconnect. (j) Liquid metal bridging patterned LIG interconnects. k) Graph showing resistance as a function of LIG width, showing tunable conductivity. l) LIG interconnect connected to an LED for logic gate output activation. m) buffer logic gate controlled via NC LIG‐based valve.

Another interesting utility of such valves for LMD sorting within a Y‐shaped channel is realized by embedding NO valves at the inlet of each branch to selectively direct LMDs based on the applied voltage. **Figure** **4**a,c demonstrate this concept, where a positive voltage is applied to valve 1 blocks the left branch, forcing the LMD to pass through the right branch. This is reversed when a positive voltage is applied to valve 2 (Figure 4c,d), preventing entry into the right branch and directing the LMD to the left branch. To maintain a uniform CEW, a shared anode is positioned at the top end of both branches of the Y‐channel. The experimental snapshots in Figure 4b,d demonstrate this sorting mechanism along with Video S6 (Supporting Information).

##### Integrated LMD Feedback Sensors

The tunable electrical resistance of LIG, its LM compatibility, and ease of patterning enable its use as a feedback sensor. In previous work, we have demonstrated that LMD can modulate LIG's resistance as it moves over it, allowing it to function as a resistive sensor.^[^ ^20^, ^46^ ^]^ Here, we integrated the same sensing mechanism for real‐time detection of LMD movement. As illustrated in Figure 4e,f, LIG‐based resistive sensors were placed sequentially along the fluidic channel immediately after each valve. As a LMD passes over a sensor, it bridges the resistive trace, lowering the resistance and detecting droplet movement. Figure 4i shows the real‐time resistance response of three sensors, each showing a resistance drop as the LMD passes. Figure 4g,h provides close‐up views of sensor‐LMD interaction. Integrating feedback sensing functionalities enhances LMD control, adding potential intelligence and closed‐loop control of LM reconfigurable systems.

#### 2.3.2 Valve‐Enabled Logic Gates

##### LIG as an Electrical Interconnect

Beyond sensing, LIG can serve as an interconnect for dynamically reconfigurable circuits with LMD acting as a movable bridge. Figure 4j,k show the electrical resistance of LIG interconnects of varying widths (W) as LMD bridges the gap between the two traces. As expected, the resistance decreases with increasing electrode width, showing the geometric dependence on conductivity. A key advantage of LIG‐based interconnects compared to metallic ones is that they provide reversible operation; LMD forms the electrical pathway at the LIG interface without leaving any residues.

##### Logic Gates

**Figure 5:**
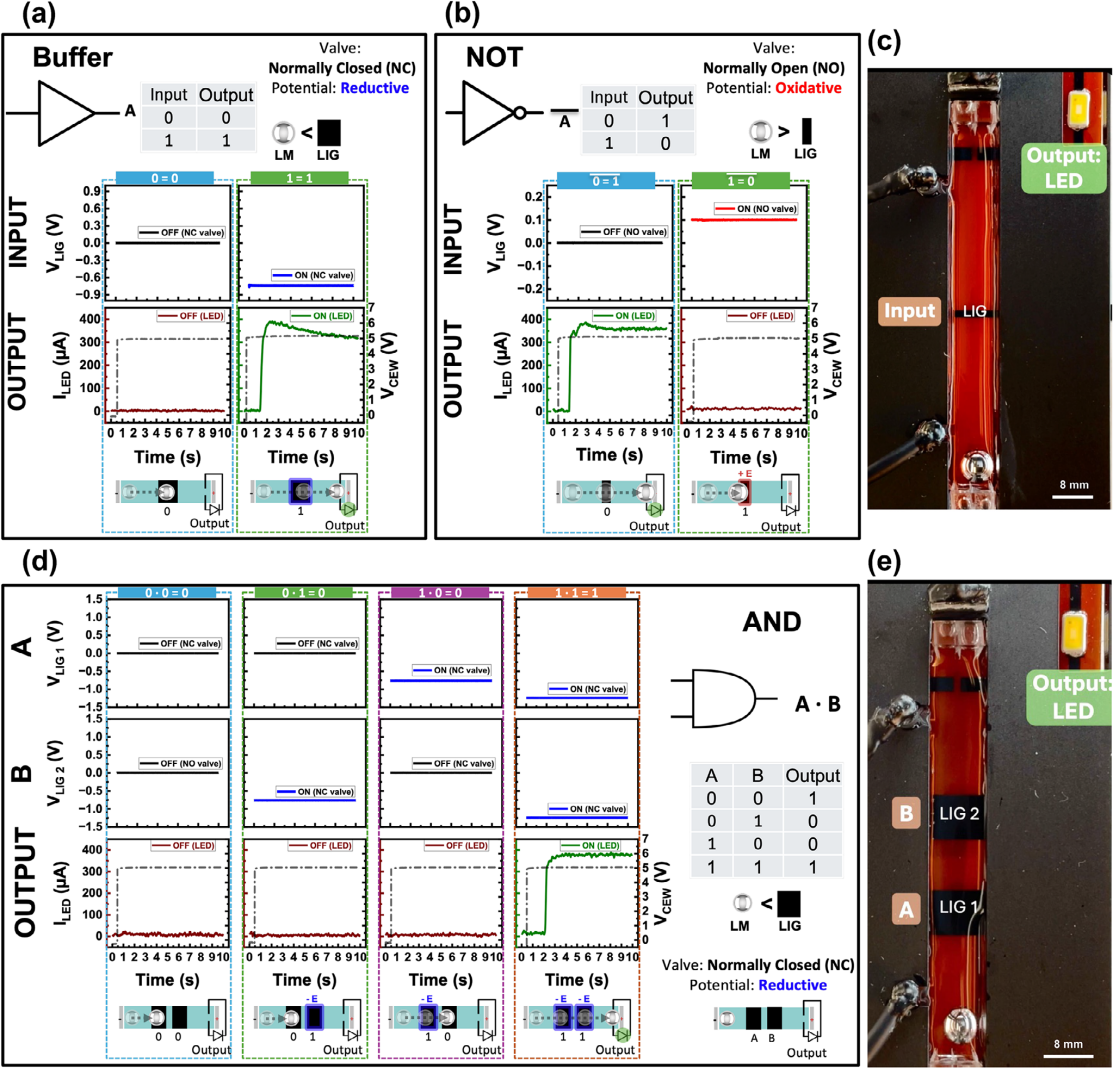
Practical applications: Logic gate implementation using LIG valves and interconnects. a,b) Single‐input logic gates: Buffer and NOT. The Buffer gate (a) uses a normally closed (NC) LIG valve, where a reductive potential opens the valve, allowing the LMD to activate the LED. The NOT gate (b) uses a normally open (NO) valve, where an oxidative potential closes the valve, switching the output. c) Image showing the NOT gate prototype. d) Two‐input AND gate using two NC LIG valves. The LMD is blocked unless both inputs receive a reductive potential, allowing it to pass and turn on the LED. Time‐series plots illustrate valve activation, LMD motion, and LED current. e) Image showing the AND gate prototype.

**Figure 6:**
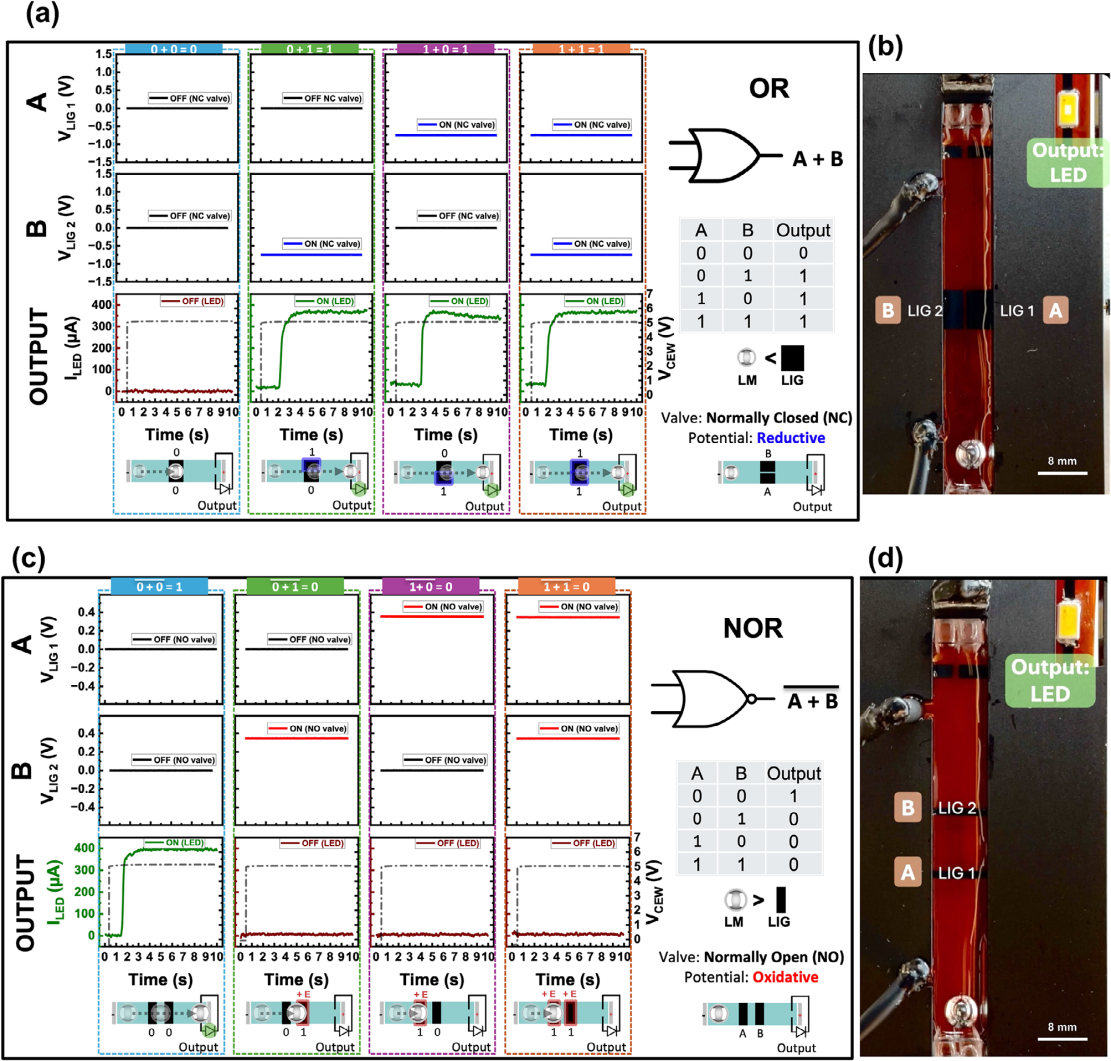
Practical Applications: Logic Gate Implementation Using LIG Valves and Interconnects. a,b) Two‐input OR gate: The OR gate uses two normally closed (NC) LIG valves, where a reductive potential applied to either input allows the LMD to pass, completing the circuit and turning on the LED. b) Image showing the OR gate prototype. c,d) Two‐input NOR gate: The NOR gate uses two normally open (NO) LIG valves, where an oxidative potential applied to either input blocks LMD motion, preventing circuit completion and keeping the LED off. d) Image showing the NOR gate prototype. Time‐series plots illustrate valve activation, LMD motion, and LED current response for both logic gates.

Utilizing LIG interconnects and valves together, we develop valve‐enabled LMD logic gates where LMD locomotion dynamically configures circuit states. When a LMD bridges two LIG traces, it completes an electrical circuit, toggling the circuit state. This is implemented by placing a light‐emitting diode (LED) with a LIG interconnect at each gate output (Figure 4l), where LMD motion determines the output state. A buffer gate prototype is shown in Figure 4m, employing a single NC valve as the input. Single‐input logic gates (Buffer and NOT) are shown in **Figure** **5**a,b. The Buffer gate utilizes a NC LIG valve, where a reductive potential opens the valve, allowing LMD to pass and switch the output LED (1 → 1) (Video S7, Supporting Information). Conversely, the NOT gate employs a NO LIG valve, where an oxidative potential closes the valve, preventing LMD from advancing through the channel and inverting the output state (0 → 1, 1 → 0) (Video S8, Supporting Information). The prototype of the NOT gate is shown in Figure 5c. For two‐input operations, AND, OR, and NOR gates are demonstrated. The AND gate (Figure 5d) is realized using two NC LIG valves arranged sequentially along the channel (prototype in Figure 5e). The LMD remains blocked unless both inputs receive a reductive potential, allowing it to pass (1,1 → 1) (Video S9, Supporting Information). The OR gate (**Figure** **6**a,b) also utilizes two NC LIG valves. However, they are arranged side by side within the channel where a reductive potential applied to either input allows the LMD to pass (Video S10, Supporting Information). On the other hand, the NOR gate (Figure 6c,d) is created using two NO LIG valves. Here, an oxidative potential applied to either input blocks LMD movement, keeping the LED off (Video S11, Supporting Information). Time‐series plots for the gates show the voltage applied to the valves (V_LIG_) (gates input), CEW voltage (V_CEW_), LMD motion, and the corresponding LED current (I_LED_) (gates output).

### 2.4 LIG‐Enabled 2D Platforms for Programmed Liquid Metal Manipulation

**Figure 7:**
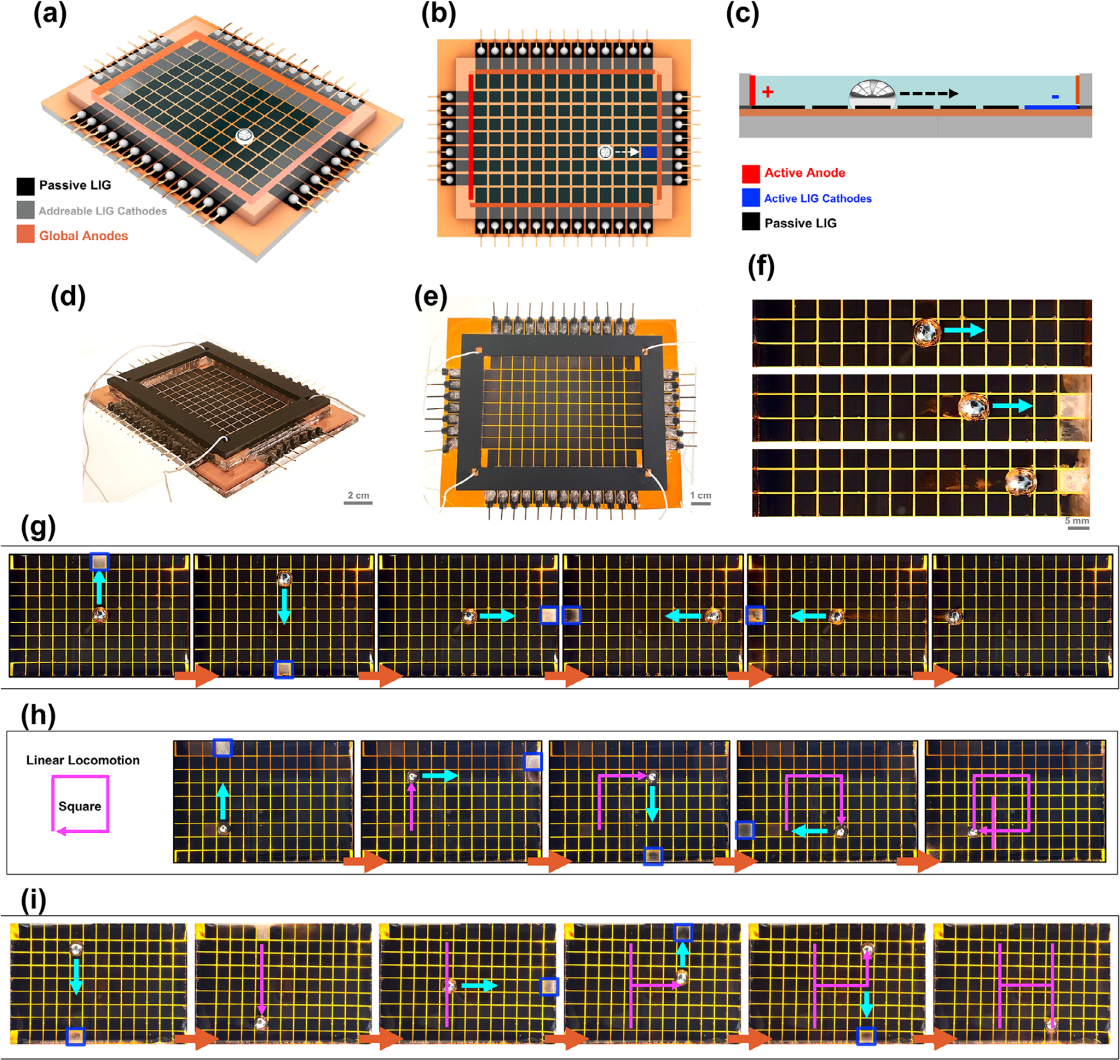
2D LIG platform for liquid metal locomotion and transformation. a–c) Schematic representation of the 2D LIG platform and control mechanism. a,b) Isometric and top–down views of the platform show passive LIG regions, addressable LIG cathodes, and global anodes. c) Cross‐sectional schematic illustrating LMD movement under electrowetting control. d–f) Photographs of the fabricated platform. d,e) Physical implementation of the system, showing integrated electrode connections. f) Sequential LMD locomotion demonstrating programmable motion across the patterned LIG surface. g–i) LMD locomotion and transformation on patterned LIG substrates. g) Controlled linear locomotion of LMD along predefined paths. h,i) LMD pattern formation, including geometric and letter tracing transformations, is achieved through sequential activation of addressable cathodes.

Building on the LM/LIG interaction, we introduce a programmable 2D LM manipulation platform that extends beyond 1D channels. This platform integrates i) a passive LIG grid/film substrate, ii) a network of addressable LIG cathodes for programmable actuation, and iii) a global anodic frame for the CEW‐driven locomotion. The 2D platform (**Figure** **7**a,b) consists of addressable LIG electrodes along all four edges, with seven on each vertical side and eleven on each horizontal side. These addressable cathodes function as programmable actuation electrodes, while the global anode frames (copper/graphite) serve as the anode to establish the CEW field with the cathodes. To direct LMD motion (Figure 7c), the cathode at the desired location is activated simultaneously with the corresponding anode on the opposite side, creating a surface tension gradient that guides its motion. LMD movement toward the cathode on LIG surfaces enables this configuration. The system is controlled via an integrated custom electronics board (Note S11, Figures S25 and S26, Supporting Information), allowing real‐time programming of each cathode to guide LMD along predefined paths in 2D. Figure 7d,e shows the fabricated platform.

#### 2.4.1 Grid LIG: Enabling Discrete LM Locomotion

As mentioned earlier, the grid LIG pattern introduces localized surface tension variation, which provides trapping sites stabilizing the LMD shape as it moves once the voltage is turned off, rather than continuously elongating. Figure 7f shows real‐time locomotion, where LMD moves to predefined locations while preserving its discrete shape toward the activated cathode (indicated by hydrogen bubbling). Sequential right‐left and up–down movement is shown in Figure 7g, with the active cathode highlighted in blue. Figure 7h,i showcases complex motion programming, where LMD traces a square pattern (7h) and forms the letter “H” (7i) through sequential electrode activation. Locomotion experiments were conducted using a CEW voltage of 12 V, provided by a DC power supply (Video S12, Supporting Information). This demonstrates the platform's potential for reconfigurable circuits and digital microfluidics.

#### 2.4.2 LIG Film: Enabling LM Elongation

**Figure 8:**
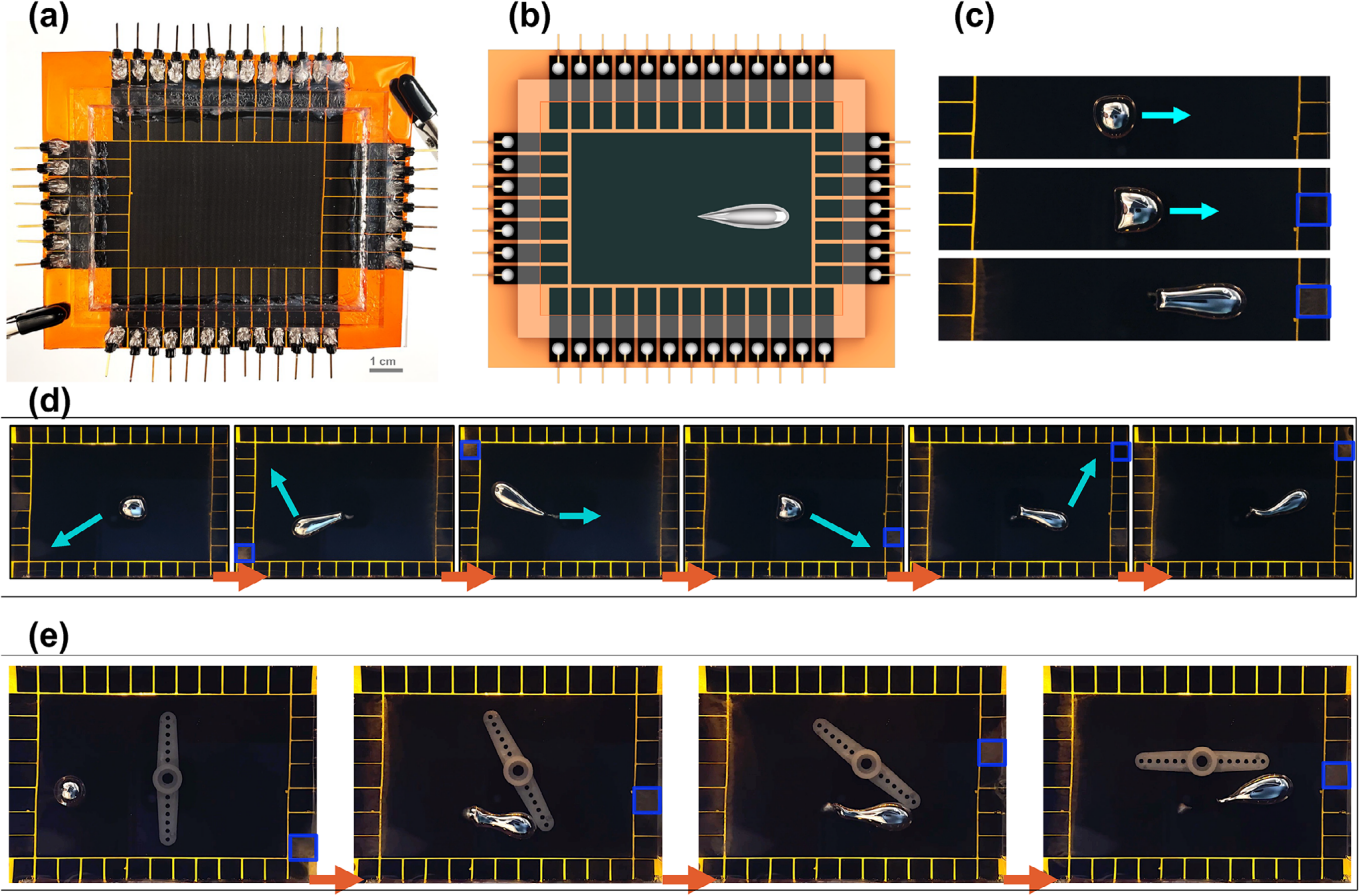
Liquid metal manipulation on LIG film. a,b) Fabricated 2D LIG platform for liquid metal manipulation. (a) Photograph of the assembled system. (b) Schematic showing LIG film as the substrate, allowing liquid metal to elongate under applied electric fields. c) Sequential snapshots demonstrate the elongation of the liquid metal droplet (LMD) on LIG film as it moves under controlled actuation. d) Directional control and diagonal motion of LMD. By selectively activating different cathodes, the LMD is guided along diagonal paths, demonstrating controlled movement and elongation in response to applied potentials. e) LM‐guided mechanical interaction. The LM is directed to push a rotational arm (or lever‐like structure), showcasing its ability to exert mechanical force on external objects. This is achieved by selectively activating the addressable outer cathodes to achieve the desired motion.

Beyond discrete LMDs, continuous LIG films enable LM stretching, as shown in **Figure** **8**a. Unlike grid‐patterned LIG, LIG films allow LM to elongate as it moves due to continuous oxidation, leading to a gradual stretching effect (Figure 8b). Figure 8c shows this elongation as LM moves toward the right‐actuated cathode. This interaction introduces strategies of shape modulation, dictated by substrate design and electrochemical effect. Additionally, LM movement is not restricted to linear paths; selective cathode activation enables diagonal motion, expanding programmable trajectories (Figure 8d). Elongation‐driven motion allows LM to exert force on external objects, as demonstrated in Figure 8e, where the LM pushes a lever‐like structure. This capability positions LM as a dynamically controlled electromechanical actuator for soft robotics and micro‐actuation systems (Video S13, Supporting Information).

#### 2.4.3 Reconfigurable Circuits

**Figure 9:**
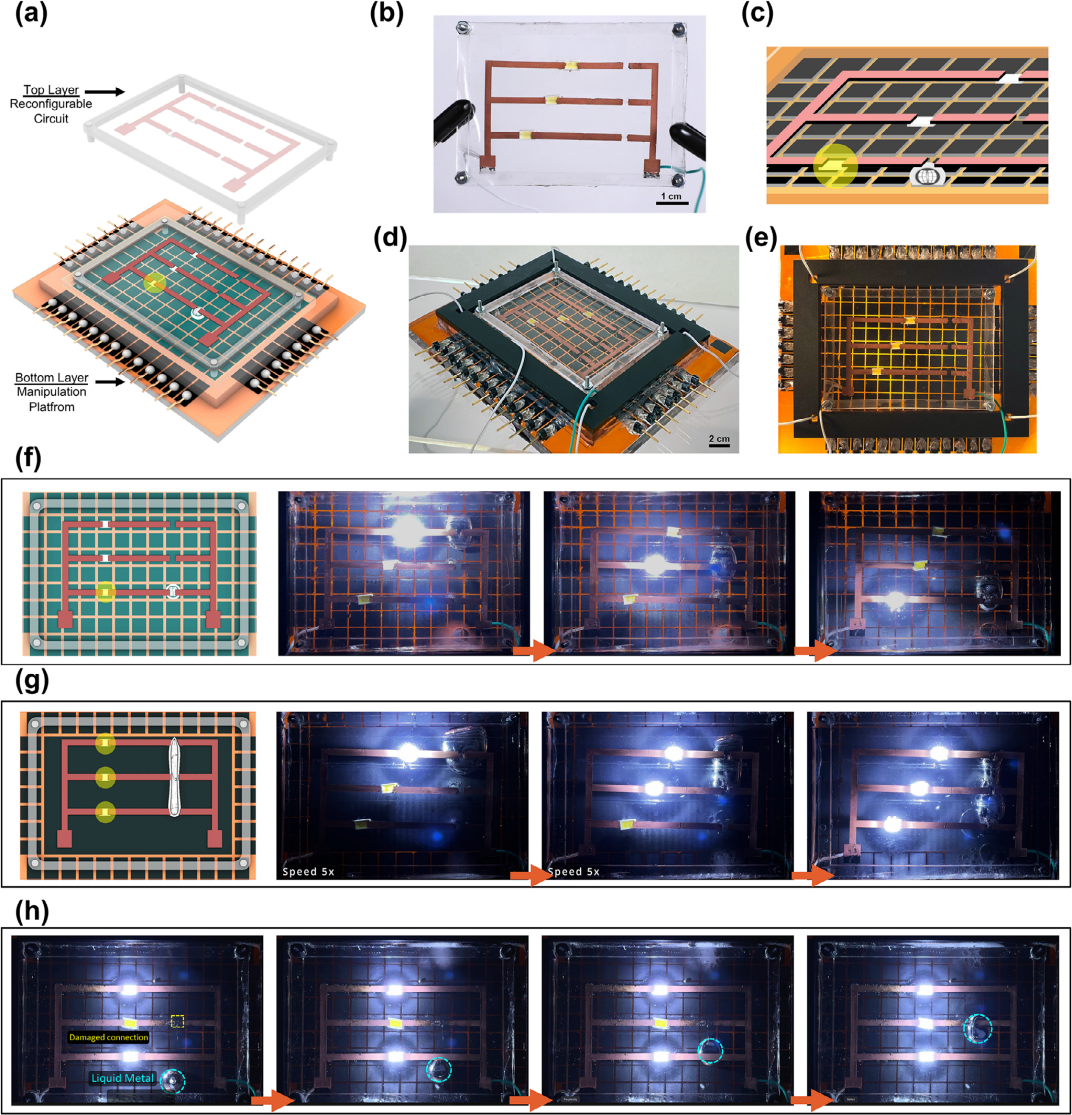
Reconfigurable circuit platform. a) Schematic of the platform design, consisting of a bottom layer with a grid‐patterned LIG manipulation platform and a top layer with copper‐plated LIG interconnects forming the reconfigurable circuit. b) Photograph of the top circuit layer. c) Schematic showing the top circuit layer, where liquid metal droplets contact the interconnects to modify the circuit configuration. d,e) Photographs of the fabricated platform, showing d) an isometric view of the assembled two‐layer system and e) a top–down view highlighting liquid metal positioning relative to the interconnects. f) Sequential LED activation via programmable LMD motion, where the LMD is guided to selectively bridge different interconnects, activating LEDs one at a time. g) Simultaneous LED activation using liquid metal elongation on LIG film, enabling the LMD to bridge multiple interconnects at once. h) Self‐healing circuit demonstration, where a damaged interconnect is repaired by guiding the LMD to the broken site, restoring electrical continuity, and reactivating the LED.

Expanding upon the programmable 2D LM manipulation, we introduce a reconfigurable electronics platform that enables real‐time circuit reconfiguration. This platform consists of a bottom layer for programmable LM locomotion using either patterned LIG or a film of LIG and a top layer with copper‐plated LIG interconnects (**Figure** **9**a). LM serves as a programmable interconnect, selectively bridging conductive paths on the top layer, and reversibly altering circuit function on demand. Unlike fixed interconnects, this platform leverages LM's fluidic and conductive nature to provide real‐time, adaptive routing. The LIG interconnects are copper‐plated on one side for improved conductivity, while keeping the LIG side interfacing with LM. The top layer has predefined interconnects, which LM can dynamically bridge, modifying circuit connectivity in response to controlled actuation (Figure 9b,c). The bottom‐layer platform precisely guides LMDs toward specific interconnects, enabling selective circuit modulation (Figure 9d,e). We showcase three reconfigurable circuit scenarios. First, LM is guided across the platform to sequentially bridge different LIG interconnects, leading to sequential activation of LEDs one at a time (Figure 9f). The second scenario demonstrates parallel LEDs activation, where LM‐controlled elongation on LIG film results in the simultaneous bridging of multiple interconnects (Figure 9g). Stretching the conductive matter enables multi‐node circuit connectivity without additional wiring. Lastly, LM can be guided to repair damaged sites by bridging interconnects and restoring electrical continuity without external intervention (Figure 9h; Video S14, Supporting Information). This self‐healing capability is particularly useful for systems in remote settings. The integration of dynamic LM manipulation with LIG‐based circuits introduces new possibilities for adaptive, reconfigurable, and self‐healing electronics.

## 3 Conclusion

This work introduces programmable continuous electrowetting (CEW) actuation of liquid metal (LM) using electrochemical modulation of the LM. By controlling the potential applied to the LM using laser‐induced graphene (LIG), the direction of LMD movement can be switched. Utilizing LIG, we developed a range of devices for LM manipulation in 1D and 2D spaces. Surface‐enabled LIG “valves” allow LMD control, sorting, and feedback sensing within fluidic channels. Extending this concept, we implemented fluidic logic gates, achieving logic operations (NOT, Buffer, AND, OR, NOR) with LMD as a responsive conductive element. A programmable 2D platform was demonstrated, enabling both discrete droplet locomotion and LM elongation via patterned and continuous LIG films. Finally, a reconfigurable circuit platform was demonstrated, where LM functions as a dynamic interconnect, for sequential and parallel circuit activation, as well as self‐healing capabilities. The approach relies on globally applied fields, limiting simultaneous control of multiple LMDs. Future efforts will focus on localized field control, where all LIG electrodes within the grids will act as individually addressable electrodes to apply small potentials for independent manipulation of multiple LMDs, including merging and splitting. Increasing electrode density and miniaturization could enhance actuation resolution, while exploring alternative LIG patterns may demonstrate new functionalities. Potential applications of the work introduced include programmable soft matter, reconfigurable and self‐healing circuits, radio frequency devices, microfluidic logic, microelectromechanical actuators, and micro‐robotic systems.

## 4 Experimental Section

### Materials and Fabrication

Eutectic Gallium–Indium (EGaIN) liquid metal with a melting point (mp) below room temperature (15.7 °C) was purchased from Sigma–Aldrich (St. Louis, Missouri, USA), composed of 75.5 wt% Ga and 24.5 wt%. The electrolyte used in all experiments was a 1 m sodium hydroxide (NaOH) solution, purchased from Sigma–Aldrich (St. Louis, Missouri, USA). The desired LMD droplet diameter was measured using top‐view optical imaging. LIG elements were patterned via direct laser irradiation of a commercial polyimide (PI) Kapton film (thickness: 127 µm) with a silicone back adhesive (CS Hyde, Lake Villa, IL, USA) using a CO_2_ laser with a wavelength of 10.6 µm (GCC Spirit GLS‐30v). The optimized parameters were 8% output power (maximum output power 55 W), 10% movement speed, and 1500 pulses inch^−1^(Note S8, Supporting Information). For all device assemblies, graphite rods and plates (McMaster‐Carr, IL, USA) were used as reference and counter electrodes, while double‐sided copper tape was used for the copper global anodes. Reliable electrical connections to LIG electrodes were established using conductive silver epoxy (MG Chemicals, Ontario, Canada). Fluidic channels and 2D platforms were fabricated using Poly(methyl methacrylate) (PMMA)/ Acrylic sheets, laser‐cut and bonded using chloroform (for PMMA‐PMMA bonding) (Sigma–Aldrich, St. Louis, Missouri, USA) and Sil‐Poxy room temperature vulcanizing silicone (for protective coating and sealing) (Smooth‐On, Inc., Macungie, PA, USA). For reconfigurable circuit fabrication, LIG patterns were first created via laser, followed by copper electroplating in a copper sulfate solution. The electroplated circuit patterns were transferred to polydimethylsiloxane (PDMS) (Dow Corning Corp., Sylgard 184) by casting PDMS (10:1 base‐to‐curing agent ratio) over the pattern. After degassing for 10 min, PDMS was cured at 80 °C for 1 h, and the PI layer was peeled off, leaving the conductive pattern embedded within the elastomer.

### Material Characterization

The microstructure of LIG was analyzed using a scanning electron microscope (SEM) (FlexSEM 1000, Hitachi, and Quattro ESEM, Thermo Scientific). Raman spectra were acquired using a confocal Raman microscope (Apyron, WITec) with a 532 nm laser wavelength.

### Electrical Characterization

DC power supplies were used to provide the CEW voltage and the electrochemical modulation voltage. Voltage, current, and resistance measurements were recorded using data logging and acquisition systems (USB‐231 USB data acquisition (DAQ), Digilent, WA, USA).

### Electrochemical Characterization

Liquid metal displacement (shift) was characterized using a potentiostat with a three‐electrode setup in chronoamperometry (CA) mode. The reference electrode used was Ag/AgCl, and the counter electrode was a platinum (Pt) mesh. Open Circuit Potential (OCP) measurements of LIG electrodes were conducted using Ag/AgCl as the reference electrode.

### Liquid Metal Droplet Motion Analysis

LMD motion was recorded at 60 frames per second (FPS). Motion Tracking and displacement analysis were captured using Tracker Video Analysis and Modeling Tool (Open Source Physics).

## Conflict of Interest

The authors declare no conflict of interest.

## Supporting information



Supporting Information

Supplemental Video 1

Supplemental Video 2

Supplemental Video 3

Supplemental Video 4

Supplemental Video 5

Supplemental Video 6

Supplemental Video 7

Supplemental Video 8

Supplemental Video 9

Supplemental Video 10

Supplemental Video 11

Supplemental Video 12

Supplemental Video 13

Supplemental Video 14

## Data Availability

The data that support the findings of this study are available from the corresponding author upon reasonable request.
