# Supplementary material for: Programmable Continuous Electrowetting of Liquid Metal for Reconfigurable Electronics
Source: Adv Mater. 2025 Sep 15;38(2):e06383. doi: 10.1002/adma.202506383 (PMC12783986; doi:10.1002/adma.202506383)
Supplement: Supplementary file 1 — Supporting Information [file ADMA-38-e06383-s015.docx]

# Supporting Information

**Programmable Continuous Electrowetting of Liquid Metal for Reconfigurable Electronics**

*Wedyan Babatain*, Christine Park, Deiaa M. Harraz, Ozgun Kilic Afsar, Cedric Honnet, Sarah Lov, Jean-Baptiste Labrune, Michael D. Dickey* and Hiroshi Ishii**

**Note S1: Experimental Setup for Liquid Metal Shift and Modulation**

To characterize LMD dynamics within a CEW field, the device shown in **Figure S1a** was built. The CEW voltage was applied using graphite anode and cathode plates, positioned 96 mm apart at opposite ends of the channel. Modulation of the LMD potential was accomplished by using a copper working electrode (WE) wetted to the LMD, which was inserted through the bottom of the channel in the middle. A graphite rod, acting as the counter electrode (CE), was placed along the positive y-axis relative to the WE. To measure the critical potential (ΔE critical) as a function of open circuit potential (OCP), a reference electrode (Ag/AgCl) was added to the electrochemical cell. As shown in **Figure S1b,** the counter electrode was positioned 8 mm north of the working electrode (+y direction in the xy-plane), while the reference electrode was further positioned 8 mm north of the counter electrode, minimizing interference with the CEW circuit. To quantify the directional shift of LMD when electrochemically modulated, a positive potential was first applied to the LMD, followed by applying the CEW voltage. A shift was defined as the moment when the liquid metal droplet displaced 1 mm toward the cathode after the CEW voltage was turned on. This 1 mm threshold accounts for the interplay of electrowetting-driven motion, charge redistribution, and interfacial interactions. The setup in **Figure S1c** was used to track this displacement and capture shifts across experiments. When an externally applied CEW field is present, we observe that the OCP of LM shifts, as shown in Figure 1d. This might also be because the potential of the reference Ag/AgCl electrode is also shifting because of the externally applied field.


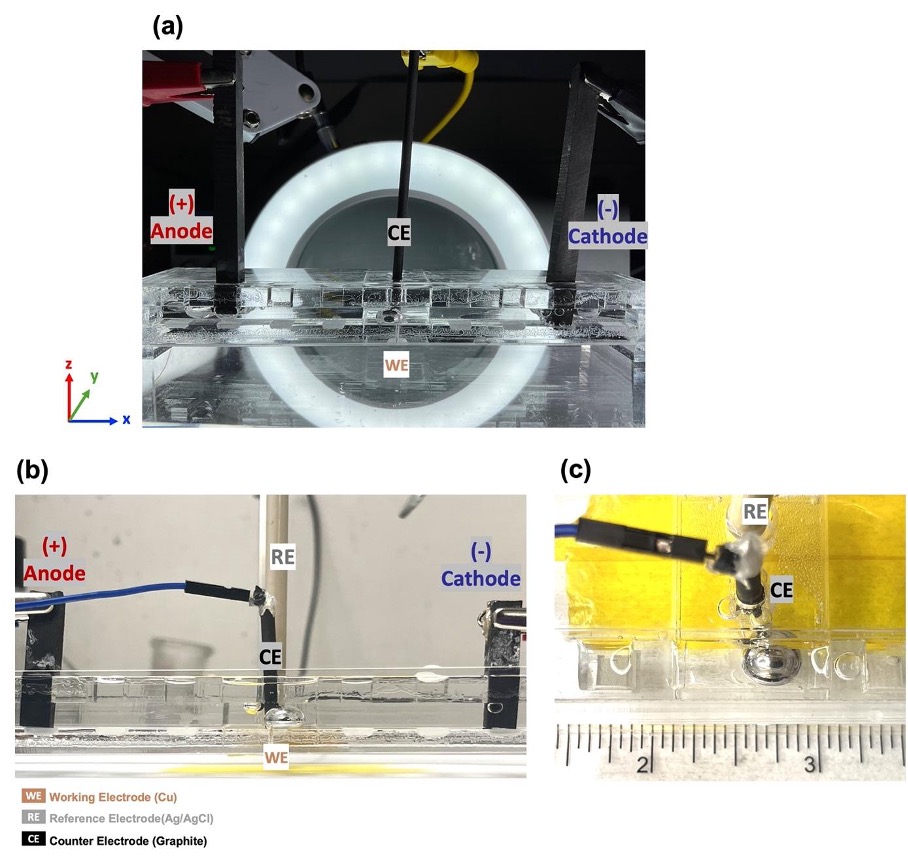


**Figure S1.** The electrochemical setup used for liquid metal characterization under CEW. (a) Photograph of the setup used for video recordings and dynamic characterization, showing an anode and cathode at opposite ends of the channel, with a working electrode (copper wire wetted to LMD) and a graphite rod as the counter electrode (CE). (b) Photograph of the electrochemical cell used for open circuit potential (OCP) and critical potential (ΔE_critical_ ) measurements, with a reference electrode (Ag/AgCl) in addition to the working and counter electrodes. (c) Top view showing the setup used to define LMD shift during directional reversal, with a ruler to measure displacement.

The reported critical potential (E_critical_) at which the direction of locomotion switches directional was chosen to be represented as as a differential value (ΔE_critical_ with respect to the specific OCP) rather than the actual potential, because the open circuit potential (OCP) of the liquid metal was observed to vary depending on the applied CEW voltage in Circuit 1. Table S1 below shows the actual measured values of both the OCP and E_critical_ (each measured vs Ag/AgCl reference) as a function of the applied CEW voltage. As seen, both OCP and E_critical_ systematically shift with the external CEW field applied in Circuit 1.

Table S1. Measured open circuit potential (OCP) and critical potential (E_critical_) of liquid metal vs Ag/AgCl under different CEW voltages.

| **V_CEW_ (V)** | **OCP_LM_ vs Ag/AgCl (V)** | **E_critical_ vs Ag/AgCl (V)** | **Δ E_critical__vs OCP(V)** |
| --- | --- | --- | --- |
| **4** | **-1.525** | **-1.26** | **0.265** |
| **5** | **-1.485** | **-1.3** | **0.185** |
| **6** | **-1.465** | **-1.35** | **0.115** |
| **7** | **-1.454** | **-1.4** | **0.054** |
| **8** | **-1.418** | **-1.38** | **0.038** |
| **9** | **-1.381** | **-1.35** | **0.031** |

Current measurement data collected using the three-electrode configuration described in Figure 1 is shown in **Figure S2** below. The current-voltage response of the LM is plotted as a function of the applied potential under three different CEW voltages (7 V, 8 V, and 9 V). In each case, the current curve demonstrates a distinct transition point, which corresponds to the experimentally observed reversal in LM locomotion direction.


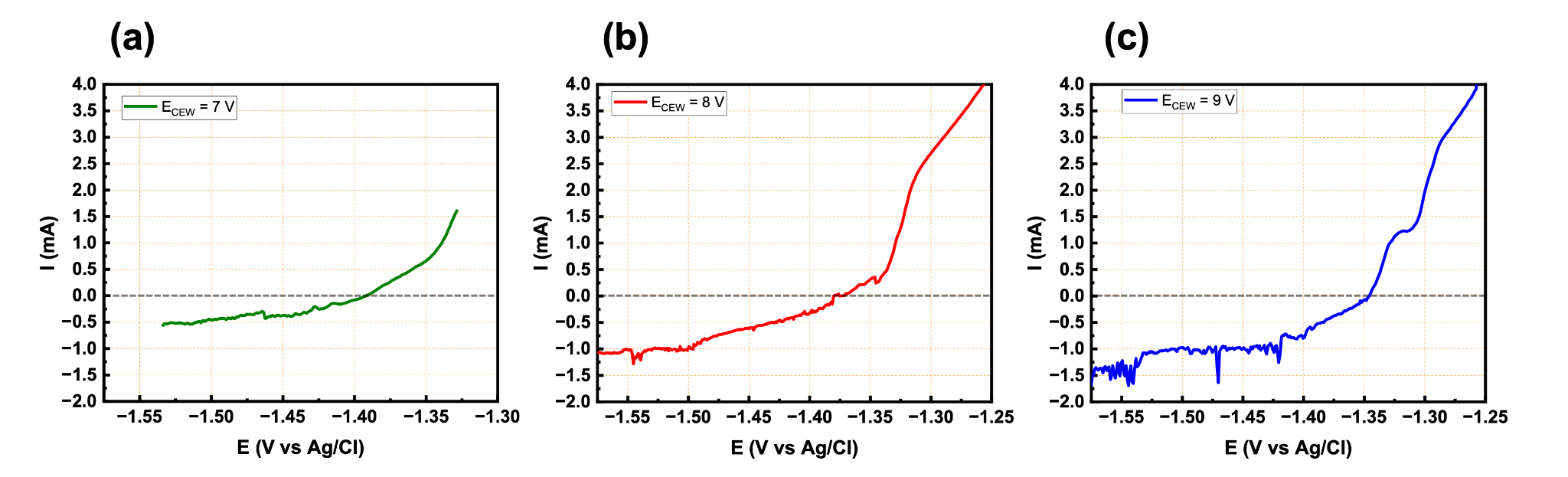


**Figure S2.** Electrochemical response curves showing current as a function of applied potential (E) vs. Ag/AgCl under different CEW voltages (V_CEW_).

**Note S2: Open Circuit Potential (OCP) Measurement of LIG Electrodes**

The open circuit potential (OCP) measurements of LIG were performed using a Ag/AgCl reference electrode. The experimental setup is shown in **Figures S3a** and **S3b** for the two states when LIG is immersed alone and when it is contacting LMD. To further examine the response of LIG OCP when an active potential is applied to it, we monitored OCP recovery after applying potential through the LIG. **Figure** **S3c** presents the relaxation behavior following the removal of a reductive potential (-1 V), where the OCP gradually returns to its baseline of approximately -0.2 V. Similarly, **Figure** **S3d** shows OCP recovery after removing an oxidative potential (+0.6 V), demonstrating the reversibility of the LIG electrode's electrochemical response. This was especially important to characterize the operation of the LIG valves.


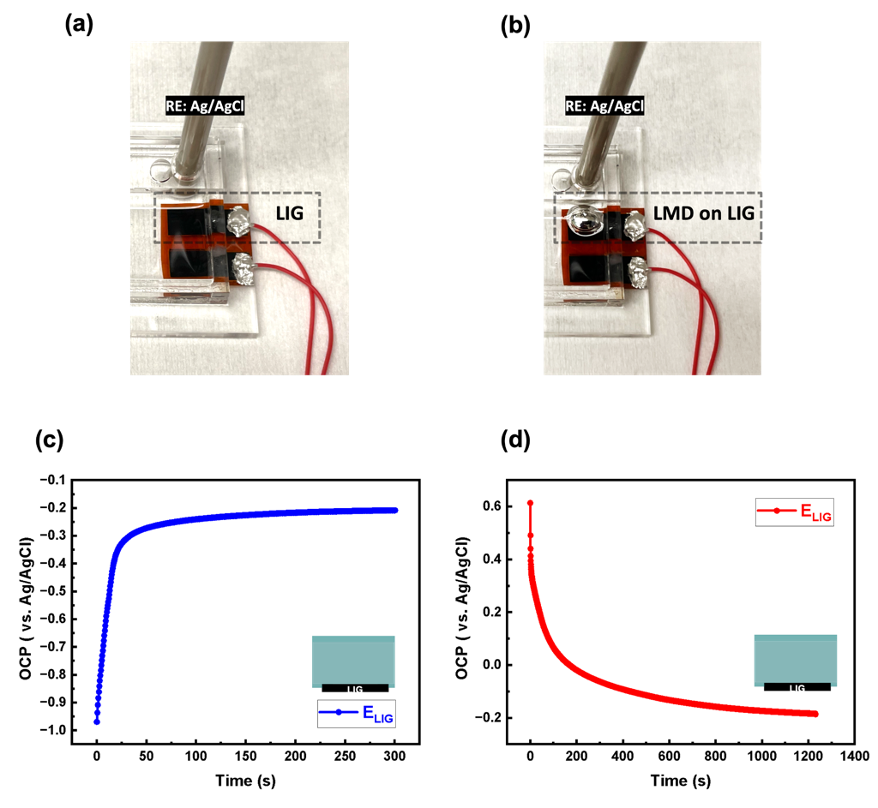


**Figure S3.** Open circuit potential (OCP) measurements of laser-induced graphene (LIG) electrodes in 1 M NaOH. (a) Electrochemical setup for measuring the static OCP of a LIG electrode immersed in NaOH, using a Ag/AgCl reference electrode. (b) When LMD makes contact with the LIG electrode. (c) Dynamic OCP recovery of the LIG electrode after removing an applied reductive potential to it (-1 V), showing relaxation back to the baseline OCP of approximately -0.2 V. (d) Dynamic OCP recovery after removing an applied oxidative potential (+0.6 V), also returning to the baseline OCP.


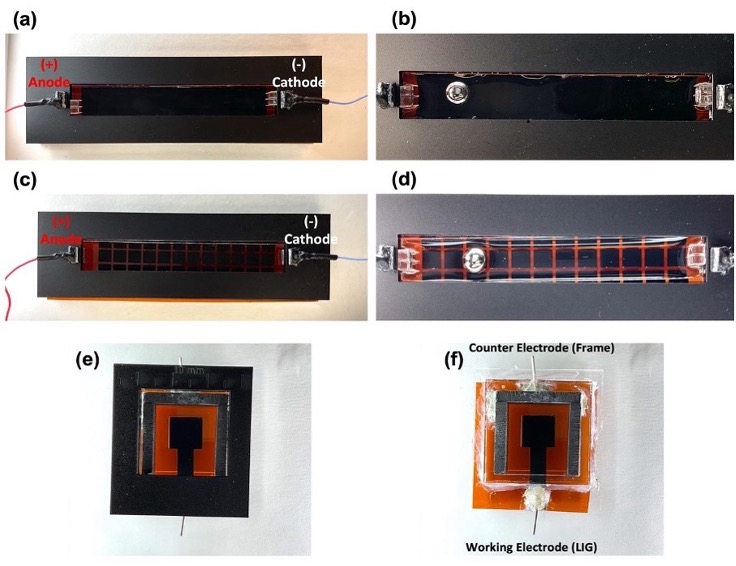


**Figure S4.** Device assemblies used for LM characterization and modulation experiments. (a-d) Photographs of the linear channel devices used for CEW characterization correspond to the experiments shown in Figure 2e-h. (a, b) LIG film channel with LMD. (c, d) Grid-patterned LIG channel used to observe discrete droplet behavior under CEW. Each device includes two graphite plates placed at the channel ends, serving as the anode and cathode. (e, f) Photographs of the device used for characterizing LIG as an active modulating electrode (Figure 2i), showing a square LIG electrode in the center with a surrounding graphite frame acting as the counter electrode. The LMD contacts the LIG electrode for modulation.

**Note S3: Continuous Electrowetting (CEW) for Liquid Metal Locomotion**

When a liquid metal droplet (such as EGaIn) is immersed in an alkaline electrolyte such as 1 M NaOH, a series of electrochemical interactions takes place. Gallium, the dominant element in LM, reacts with hydroxide ions, forming gallate species [Ga(OH)_4_]^−^ that accumulate at the droplet surface, making it negatively charged. This charge distribution attracts Na^+^ ions from the solution, leading to the formation of an electrical double layer (EDL) around the LMD as depicted in **Figure S5a**. Upon applying a DC electric field, the EDL distribution becomes asymmetric as shown in **Figure S5a**. The side of the droplet facing the anode experiences a reduction in surface tension, while the side facing the cathode retains a higher surface tension. The relationship between surface tension (γ) and the applied voltage (V) is described by the Lippmann equation:

${\gamma(V)=\gamma_{0}-\frac{1}{2}C(V-V_{0})}^{2}$ ^[2]^  (1)

Where $\gamma$ is the surface tension of the LMD, V is the electrical input, $\gamma_{0}$ is the maximum surface tension at $V_{0}$= 0, $C$is the capacitance of the EDL

Since liquids preferentially spread in regions of lower surface tension to minimize surface energy, the liquid metal wets more toward the side facing the anode. This creates a surface tension gradient, breaking the symmetry and propelling the droplet toward the anode under CEW. This mechanism explains the well-established CEW-driven motion of liquid metal on non-graphitic substrates (e.g., polyimide or glass) ^[1,2]^. When the LMD is placed on a LIG substrate immersed in an electrolyte, its motion under an applied CEW field exhibits a reversal in direction compared to conventional CEW on non-graphitic substrates as seen in **Figure S5b**. While LM typically moves toward the anode in a basic electrolyte due to a surface tension gradient, we observe that LM on LIG instead moves toward the cathode. This observation suggests that the underlying charge distribution and surface interactions may be fundamentally different on LIG. The reversal in motion indicates a modification in the surface tension gradient across the LM droplet, which is likely influenced by charge redistribution at the LM-LIG interface. Physically, LM in contact with LIG appears to undergo oxidation, as evidenced by visible changes in its surface tension and spreading behavior. This suggests a possible electrochemical interaction between LM and LIG. Furthermore, open circuit potential (OCP) measurements indicate a shift when LM is in contact with LIG, which is consistent with this model of an electrochemical interaction. Based on these observations, we hypothesize that charge redistribution occurs at the LM-LIG interface, modifying the electric double layer (EDL) and altering the surface tension gradient. As a result, the surface tension on the side of the LM closer to the cathode may be lower than that on the anode-facing side, creating a gradient that drives motion toward the cathode.


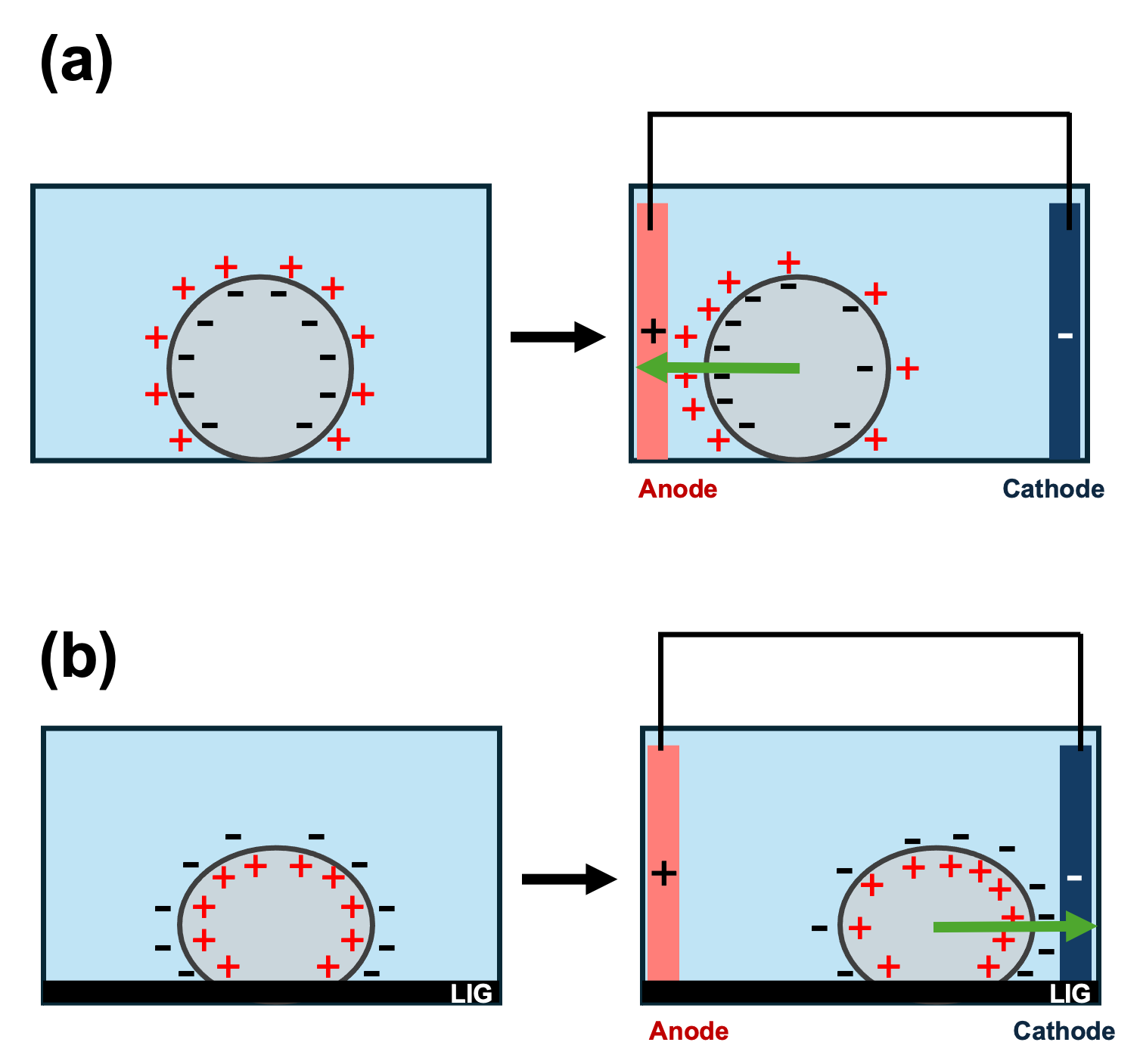


**Figure S5.** Influence of passive LIG substrates on LMD locomotion under CEW. a) Schematic showing LMD locomotion on a polyimide (non-graphitic) substrate in 1 M NaOH, where the droplet develops a negative surface charge and moves toward the anode. b) Schematic showing LMD movement on a LIG film substrate, where the droplet surface charge is altered and moves toward the cathode.

**Note S4: Electrochemical Modulation of Liquid Metal Using LIG Electrode**

**Figure S6** shows the mechanism by which LIG functions as an active modulating electrode to control the interfacial tension of LMD on demand. This process leverages the principles described by Khan et al. (2014) ^[3]^, where the surface tension of gallium-based liquid metals can be reversibly tuned via electrochemical oxidation and reduction. When an oxidative potential is applied to the LIG electrode (left panel), the liquid metal in contact with it undergoes surface oxidation, forming a thin gallium oxide layer. This oxide layer acts as a surfactant, drastically reducing the interfacial tension of the liquid metal, leading to spreading and flattening. On the other hand, when a reductive potential is applied (right panel), the oxide layer is electrochemically reduced, restoring the LMD’s high surface tension, causing it to bead up. This electrochemical modulation of surface tension allows for precise control over liquid metal morphology and locomotion. By dynamically modulating the liquid metal interface. Via LIG, we could selectively oxidize or reduce the liquid metal at the electrode interface providing an electrochemical knob for programmable liquid metal movement.


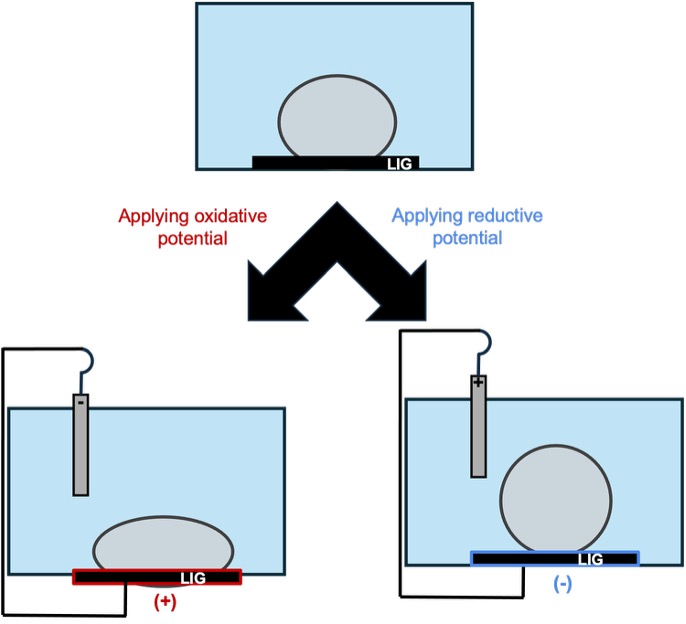


**Figure S6.** Liquid metal (LM) modulation using LIG as an active electrode. Left: Schematic showing the effect of applying an oxidative potential to the LIG electrode, inducing oxidation, reducing the LMD surface tension, and causing it to flatten. Right: Schematic showing the effect of applying a reductive potential to the LIG modulating electrode, inducing reduction and increasing the LMD surface tension, causing it to bead up.

**Note S5: Durability of LIG Electrodes Under Extended Liquid Metal Motion**

Gallium-based liquid metals are indeed known to interact corrosively with many metals, such as aluminum ^[12]^, through alloying or surface reactions. However, this is not the case for LIG or graphitic materials in general ^[5,6]^. Our experiments confirm that no corrosive degradation occurs due to LM contacting liquid metal, even under extended contact. Nonetheless, the reviewer raises an equally important point: even if no chemical corrosion occurs, the repeated mechanical contact between the LMD and the LIG substrate during extended operation could result in physical degradation of the LIG including possible flaking, delamination, or changes in LIG conductivity. Assessing this aspect is critical for evaluating long-term system reliability.

To investigate this, we performed a systematic mechanical cycling test, designed to simulate long-term usage and dynamic LMD motion over LIG. As shown in **Figure S7a**, we fabricated three 3 mm × 10 mm LIG electrodes positioned at the bottom of a 50 mm-long channel. Using continuous electrowetting (CEW) actuation of 7 V, with graphite cathodes and anodes placed at the two ends of the channel, we allowed the LMD to continuously move back and forth between the electrodes. To control the cycling, we used an H-bridge connected to the power supply to reverse the polarity. Each complete cycle (forward and backward movement) lasted 3 seconds, and the test operated for over 9 hours, totaling 10,000 cycles of continuous movement. The test allowed us to evaluate the mechanical and electrical robustness of the LIG substrate electrodes under repeated LMD movement.

**Figure S7a** shows the experimental setup and the three electrode strips and representative snapshots of the first full cycle. Figure S7b shows the tracked displacement profile of the LMD for the first 1,000 cycles, used as a representative window of the repetitive LMD movement in the experiment. Continuous tracking over the full 9-hour test was impractical, but motion patterns remained consistent throughout. Zoomed-in plots of the cyclic movement of the LMD is shown at different windows in **Figure S7c**. The experiment was paused at defined intervals (250, 500, 1,000 cycles, then every 1,000 thereafter) to measure the electrical resistance of each LIG strip.


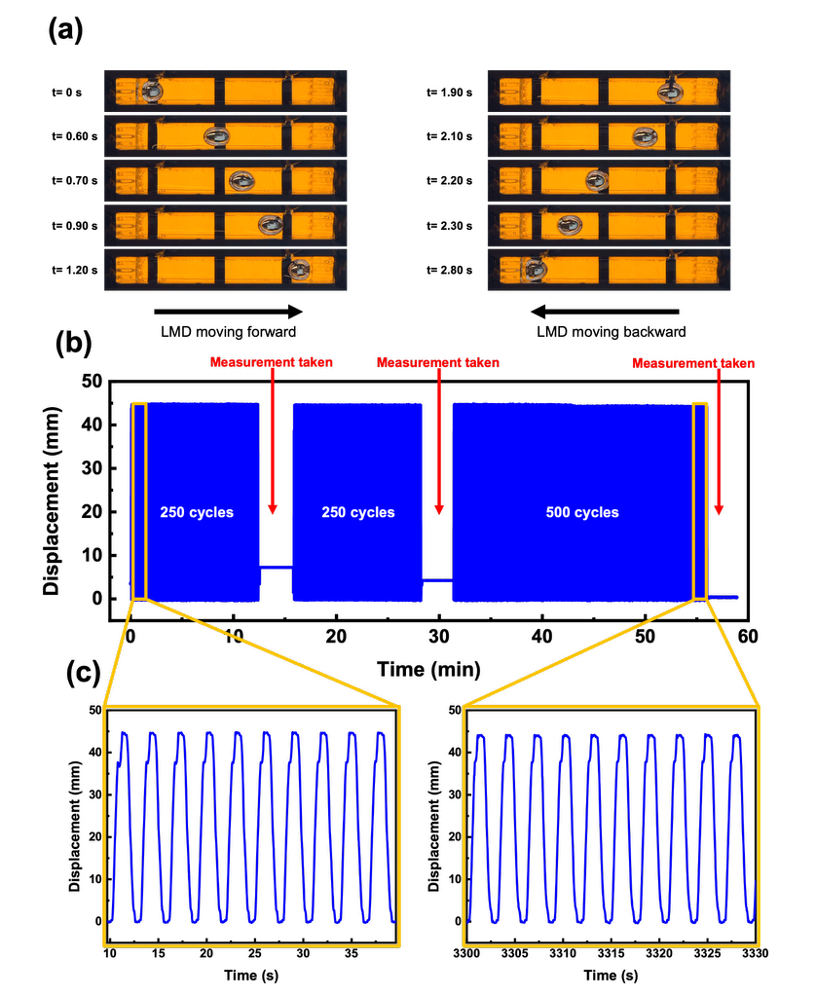


**Figure S7.** Long-term dynamic cycling test to assess the mechanical and electrical durability of LIG under continuous LMD movement. (a) Experimental setup showing LMD cycling on three parallel LIG strips under 7 V CEW between fixed graphite electrodes. (b) Sequential droplet displacement during cyclic actuation (one cycle = 3 s back-and-forth) for the first 1,000 cycles, with intermittent pauses for resistance measurements after 250, 500, and 1,000 cycles. (c) Zoomed-in displacement profiles for selected intervals showing consistent cyclic behavior.

As shown in **Figure S8a,** resistance remained stable for all three electrodes, indicating no loss in conductivity or material degradation. Minor fluctuations, both increases and decreases, in resistance were occasionally observed, likely due to transient electrochemical interactions with the LMD during the pause period, as measurements were taken 60 seconds after pausing the actuation. To assess mechanical durability, optical microscope images were captured before and after the full 10,000-cycle test (**Figure S8b**). No visible damage, delamination, or wear was observed. The white patches seen in the after-test images are attributed to residual NaOH salt deposits after the electrolyte dried.

Collectively, these results demonstrate the long-term electrical and mechanical stability of LIG electrodes under continuous LMD motion, supporting their suitability as electrode and substrate material of the developed reconfigurable systems.


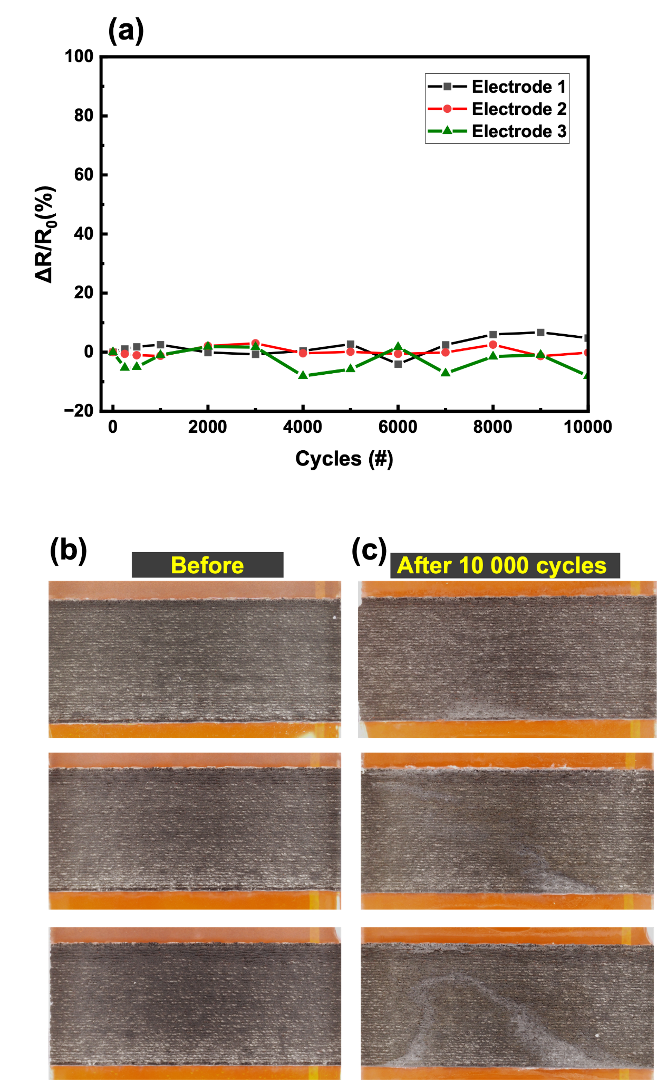


**Figure S8.** Relative resistance change (ΔR/R₀) of three LIG electrodes measured periodically throughout the 10,000-cycle test, showing minimal fluctuation and stable electrical performance. (b) Optical microscope images of each electrode before testing. (c) Corresponding images after 10,000 LM movement cycles, revealing no visible surface damage, delamination, confirming excellent mechanical resilience of the LIG electrodes.


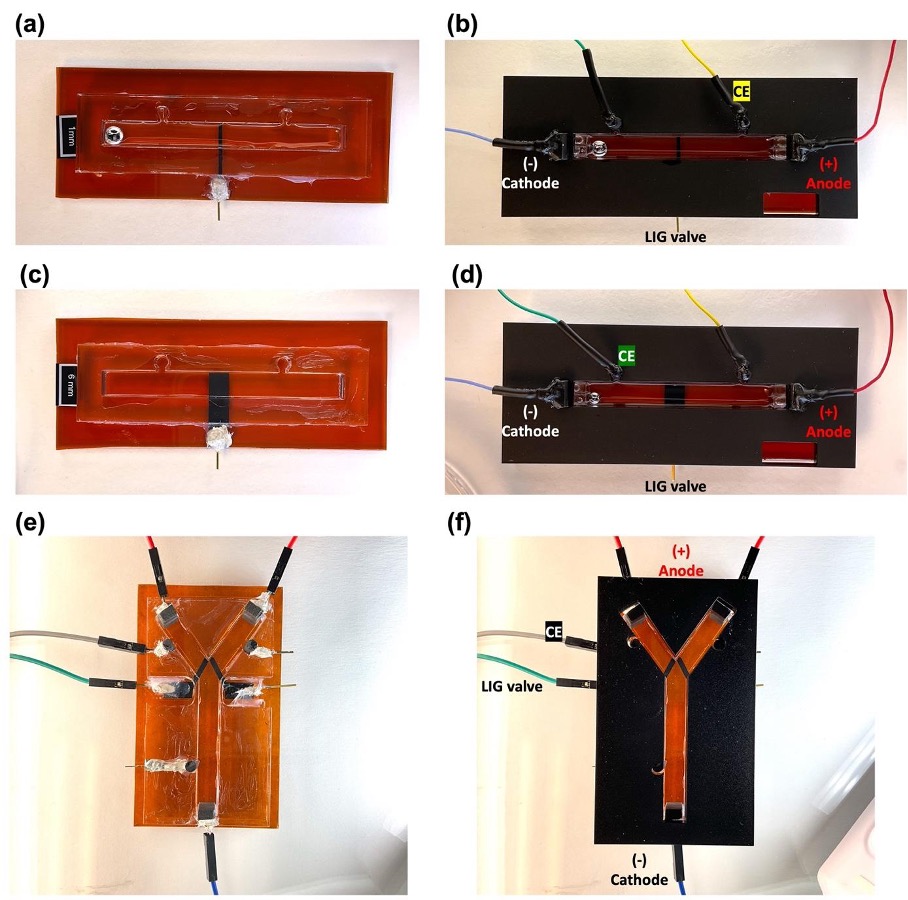


**Figure S9.** Device assemblies for LIG valve and sorting experiments. (a, b) Normally open (NO) valve. (c, d) Normally closed (NC) valve. (e, f) Y-shaped channel for LMD sorting. All devices show the placement of the valve electrode, graphite counter electrode, and CEW anode and cathode.


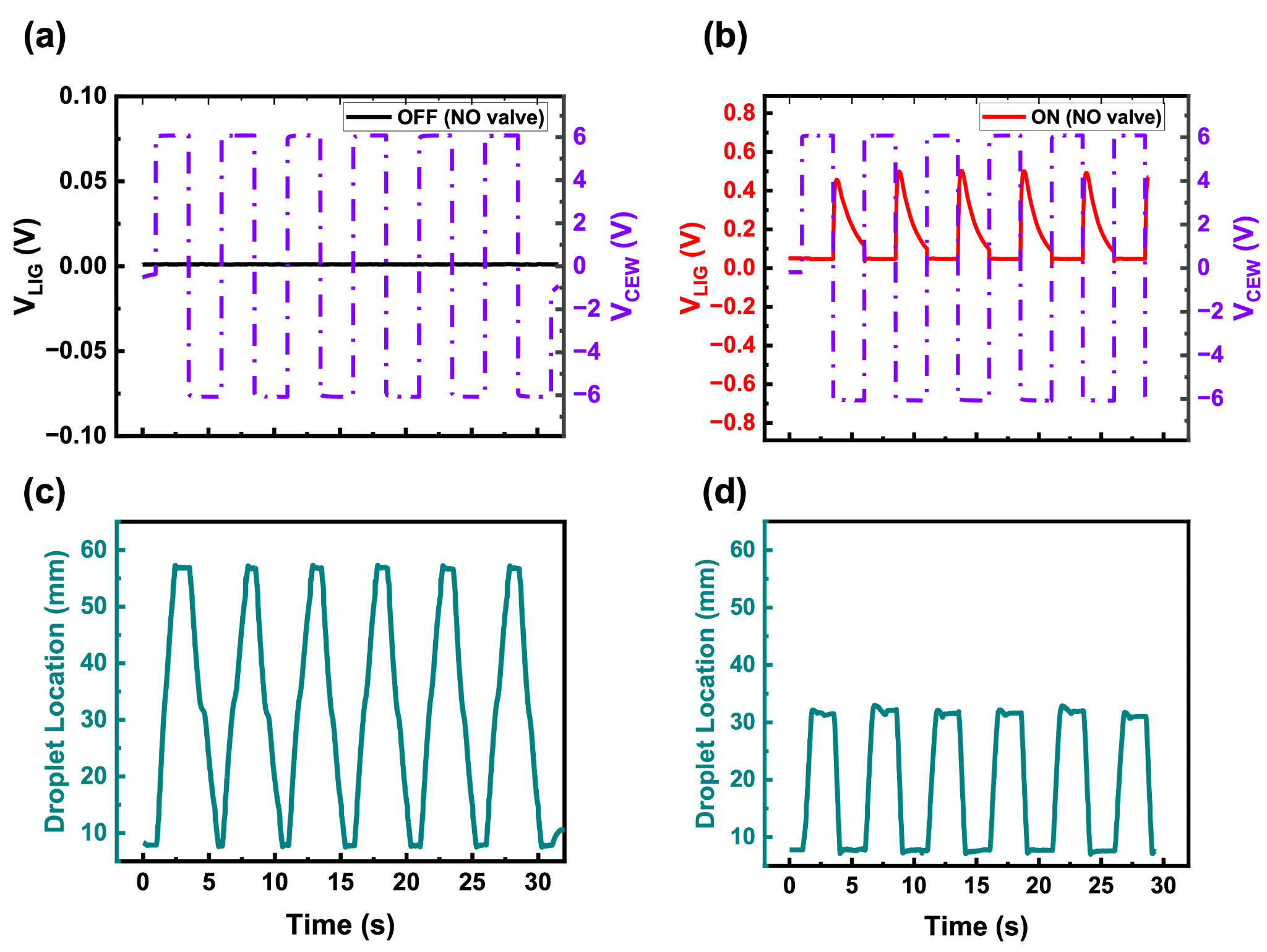


**Figure S10.** Cyclic operation of a normally open (NO) LIG valve over 6 cycles. (a, b) Applied LIG potential (V_LIG_), CEW voltage (V_CEW_), and LMD displacement for the valve in open (a) and closed (b) states. (c, d) LMD displacement profiles for open (c) and closed (d) states. Cycles show restricted motion when the valve is closed.

**Table S2.** Optimization results for the operational range of passive normally open (NO) valves (1 & 3 mm widths). LMD passage and blockage are shown as functions of size ratio and CEW voltage magnitude.

**
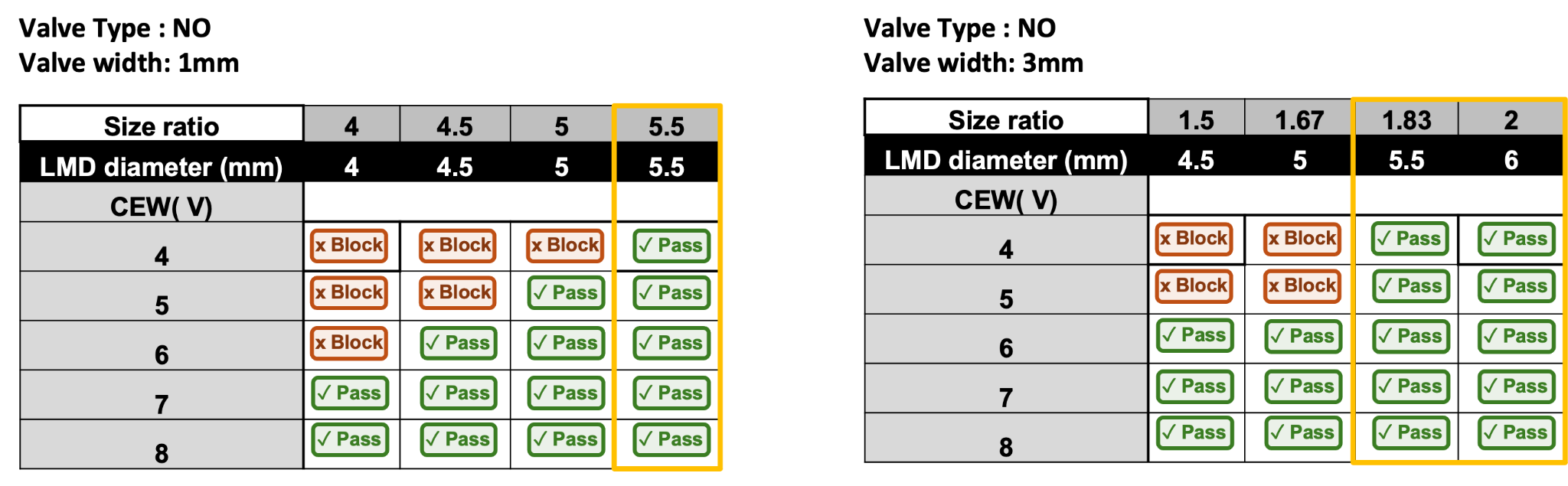
**

**Table S3.** Optimization results for the operational range of passive normally closed (NC) valves (6 & 8 mm widths). LMD passage and blockage are shown as functions of size ratio and CEW voltage magnitude.

**
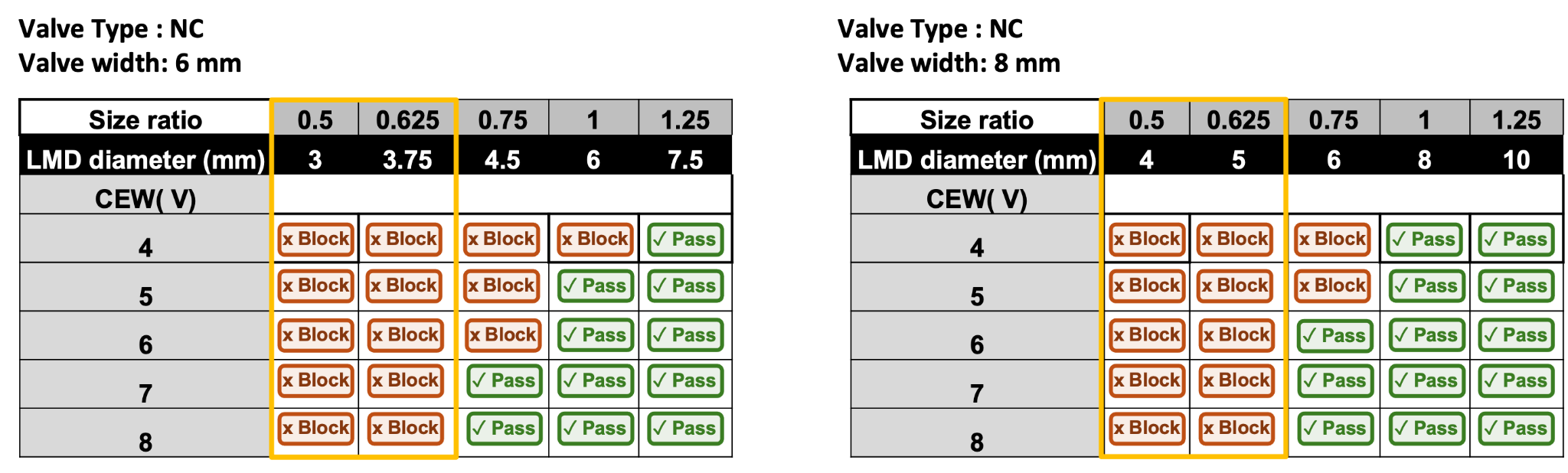
**

**Note S6: Interference Between the Continuous Electrowetting Field and Local Electrochemical Field**

Given the unique setup of our system, and the fact that we have two fields, the large continuous electrowetting field applied externally (circuit 1), and the small electrochemical field applied directly to the liquid metal droplet (circuit 2), as shown in Figure S11a, each with its own power supply. Field 1 is a global electrowetting field applied between an external anode and cathode to induce continuous liquid metal locomotion, while Field 2 is a localized electrochemical potential applied directly to LMD to modulate its interfacial tension through a working electrode (WE) and counter electrode (CE). During our experiments, we observed that the presence of the two fields within the same electrolyte resulted in an interference effect that influenced the electrochemical oxidation of the liquid metal. It was observed that the placement of the counter electrode of field 2 strongly affects the effective modulation of LMD by field 2. In some cases, the interference enhances the oxidation occurring at the WE of field 2, and in some cases, it largely diminishes it. To investigate this interference systematically, we designed a controlled experimental setup to characterize how Field 1 affects Field 2. The custom-built acrylic electrochemical cell that was used is shown in Figure S11b, which allows the placement of the CE at different positions along the channel. By systematically adjusting the CE location of Circuit 2, we could observe the impact of Field 1 on the local electrochemical behavior of Field 2. Figure S11c shows the predefined CE positions used for measurement, the WE is always fixed in the middle of the channel, location 11. Figure S11d presents the current distribution in the Field 2 circuit when only Field 2 is turned on, showing the baseline response at each CE position in the absence of Field 1. Figure S11e then illustrates the measured current when Field 1 is turned on, revealing how the presence of Field 1 strongly influences the response of Field 2 at each location. The differences between Figures S11d and S11e demonstrate the interference effect caused by Field 1, where certain CE positions experience enhanced current, indicating increased oxidation at the liquid metal, while others show suppressed current, corresponding to a reduction in oxidation activity. The heat map comparison confirms that Field 1 imposes a background interference across the electrolyte, which affects the local electrochemical conditions of Field 2. When the CE is positioned near the anode of Field 1( such as location 9), oxidation at the liquid metal is enhanced, resulting in a higher current in the Field 2 circuit. In contrast, when the CE is located closer to the cathode of Field 1 (such as location 13), oxidation is suppressed, leading to a lower current response. When the CE is placed outside the direct lateral path of Field 1(location 4), the interference effect is minimized.

The observed interference between the two fields can be attributed to bipolar electrochemistry effects induced by the external field. In this system, the external field (Field 1) influences the potential of the counter electrode, effectively creating a secondary bipolar system between the counter electrode and the working electrode (EGaIn)^[7]^ ^[4]^. Since the counter electrode is not directly controlled by its power supply, its potential is altered by the surrounding field, leading to variations in the current measured within the second circuit (Field 2). The inner circuit/field 2, which comprises the liquid metal and counter electrode, seems to act as a single entity due to their electrical connection. This means that as a single entity, it is experiencing a bipolar effect where oxidation occurs at one electrode (liquid metal) while reduction simultaneously takes place at the counter electrode. The external field forces this bipolar behavior, modifying the electrochemical response based on the counter electrode's positioning. Looking at LMD and the CE as one entity, when the counter electrode is placed near the anode of Field 1, the external field induces oxidation at the liquid metal and reduction at the CE, which will enhance LMD oxidation, leading to an increase in the measured current. Conversely, when the counter electrode is positioned near the cathode of Field 1, the external field induces reduction at LMD and reduction at the CE, so the oxidation of the liquid metal is suppressed, resulting in a lower current. When the counter electrode is placed outside the lateral field path, interference is minimized, and the bipolar effect primarily influences only the liquid metal, leading to a minor current increase. Additionally, electrolyte conductivity gradients could contribute to the observed interference. The external field may create localized conductivity variations in the electrolyte, altering ion transport and affecting current flow depending on the counter electrode's position. While this effect cannot be completely excluded, it is less likely to be the primary cause of the observed behavior. These findings show how the interaction between two existing fields alters the local electrochemical environment or the LMD and how understanding the bipolar nature of the inner circuit is essential for interpreting the observed current variations.


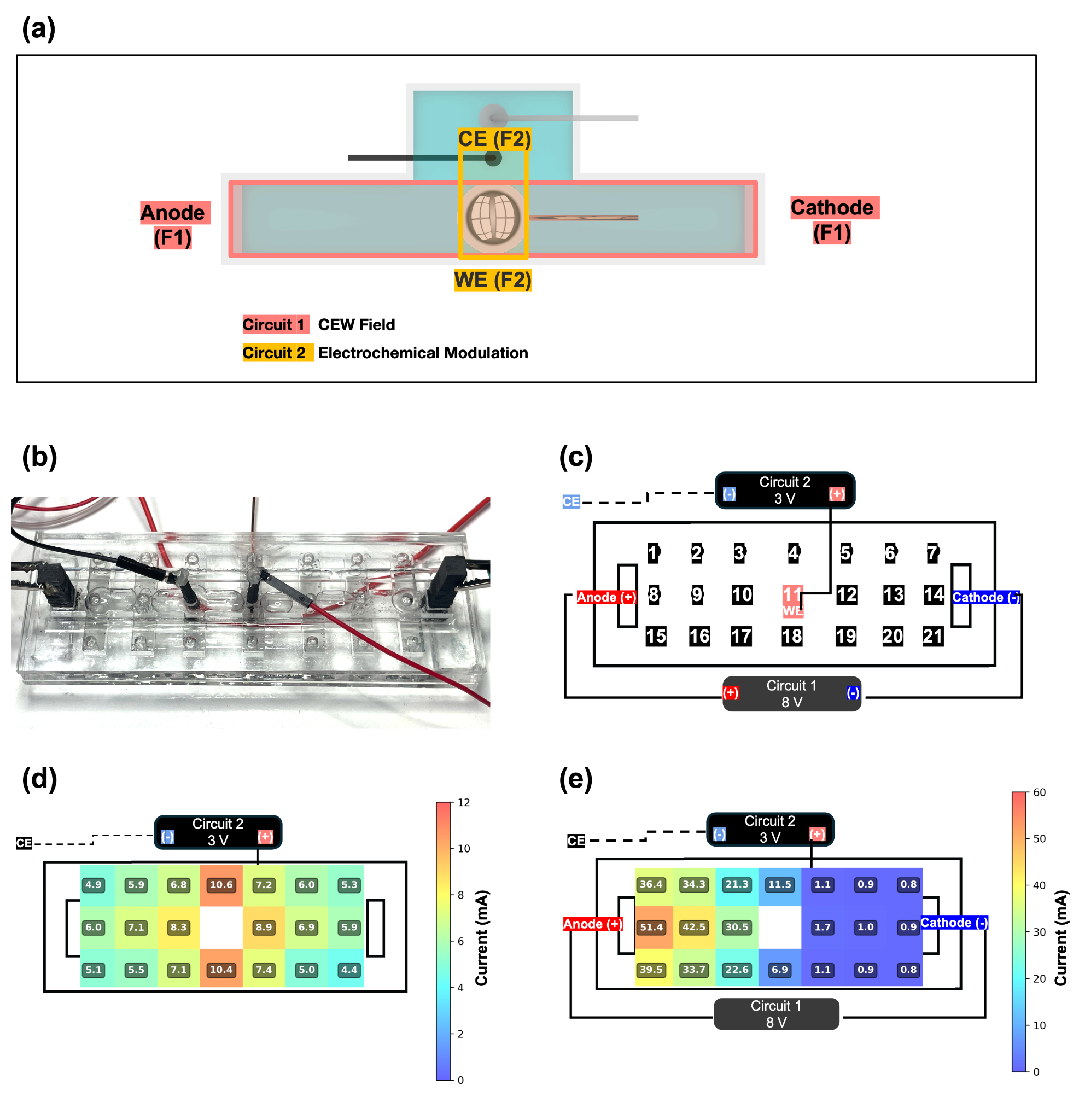


**Figure S11.** Experimental Setup and current mapping for studying interference between the CEW field and the modulation field. (a) Schematic representation of the two electric fields: Field 1 (CEW field for LMD locomotion) and Field 2 (local electrochemical field for modulation).(b) Photograph of the experimental electrochemical cell, showing fixed anode and cathode electrodes for Field 1, and a movable counter electrode (CE) for Field 2. (c) Diagram showing the tested positions of the movable CE along the channel. (d) Heatmap of the measured current in Field 2 at each CE position with Field 1 off. (e) Heatmap of the measured current in Field 2 at each CE position with Field 1 on, showing the interference effect.

**Note S7: Interference Implications for LIG-Valve Operations and CE Placement**

Understanding the interference between Field 1 and Field 2 was critical in optimizing the placement of counter electrodes for the developed valve operations. To ensure the desired electrochemical modulation at the LIG valves, the CE placement was strategically adjusted based on whether oxidation or reduction was required. For oxidative valve activation (e.g., closing a normally open valve), the CE was placed near the anode of Field 1 to enhance oxidation at the liquid metal. For reductive valve activation, the CE was positioned near the cathode of Field 1 to reinforce reduction. This strategic placement ensured reliable valve operation regardless of the present interference between the two circuits.


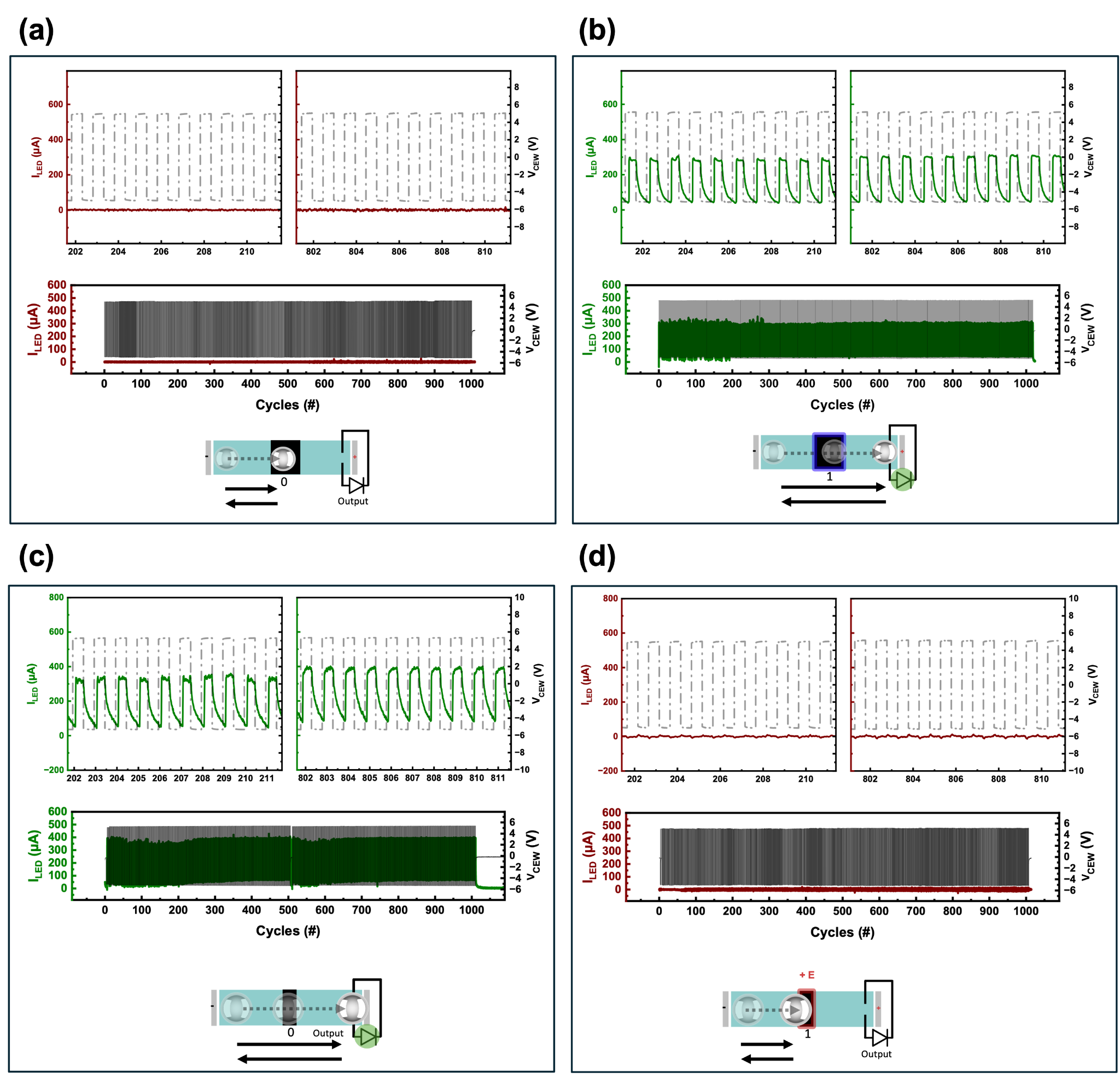


**Figure S12.** Cyclic operation of buffer and NOT gates over 1000 cycles. (a, b) Buffer gate: cycling with input = 0 (a) and input = 1 (b), showing stable LED current (I_LED) and CEW voltage (V_CEW_) over time. Insets show zoomed-in views of the first and last 10 cycles. (c, d) NOT gate: cycling with input = 0 (c) and input = 1 (d), showing stable performance over 1000 cycles, with I_LED_ and V_CEW_ monitored. Insets show the first and last 10 cycles.


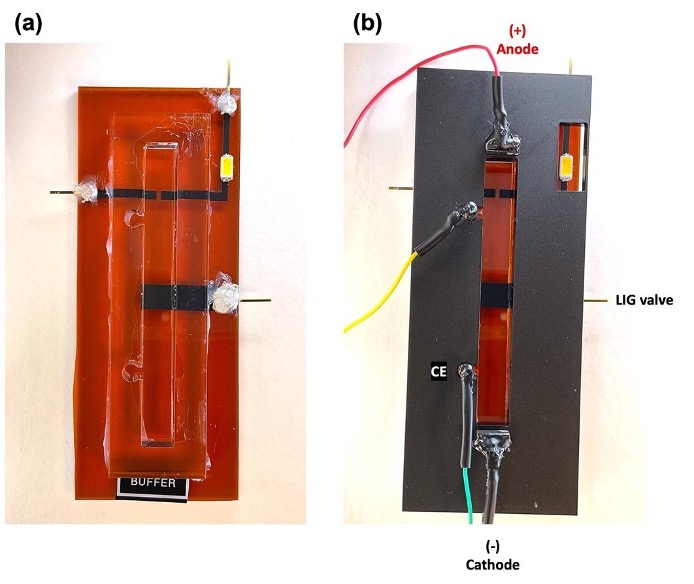


**Figure S13.** Assembly of the buffer gate platform. (a) Bare device showing the LIG valve, electrical connections, and LIG interconnect connected to the LED output. (b) Fully assembled platform with a top frame, a counter electrode, and a CEW anode and cathode at the channel ends.


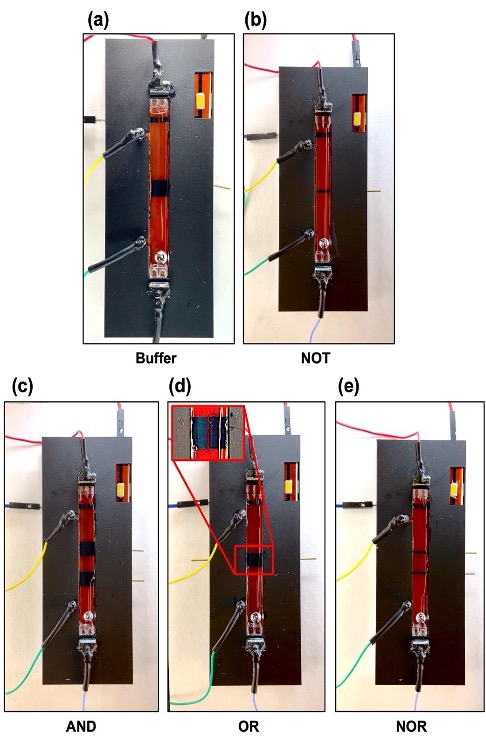


**Figure S14.** Fully assembled platforms for logic gate demonstrations. (a-e) Assembled devices for the buffer (a), NOT (b), AND (c), OR (d), and NOR (e) gates, showing electrode, valves, and output LEDs connections. Inset in (d) shows the side-by-side arrangement of two LIG valves for the OR gate.

**Note S8: Effect of Laser Power on LIG Morphology, Resistance, and Liquid Metal Locomotion**

LIG serves as the core electrode material in our system, enabling programmable actuation LMDs. To understand how laser processing conditions influence the resulting electrode performance, we systematically studied the effect of CO₂ laser power on the morphology, electrical resistance of LIG electrodes and their subsequent effect on LMD locomotion. To isolate the effect of laser power, we fixed all other fabrication parameters (e.g., laser speed, focus, pulse per inch) and varied only the laser power from 2.5 W to 6.5 W. As shown in **Figure S15**, optical images show that the optimal range for producing uniform and adherent LIG electrodes is from 2.5 W to 5.0 W. At higher powers (5.5-6.5 W), we observed significant surface damage and delamination, indicating over-carbonization of the polyimide and poor adhesion due to flaking of the LIG.


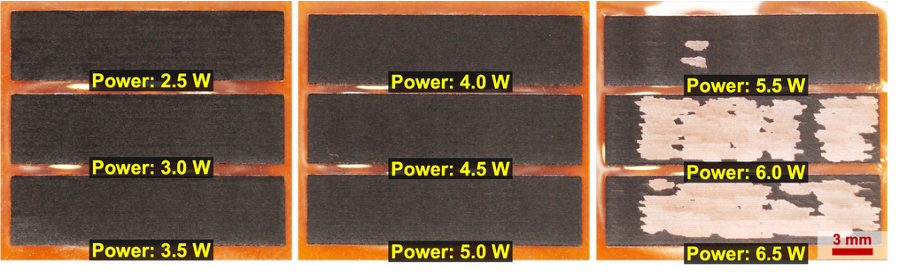


**Figure S15.** Characterization of laser-induced graphene (LIG) electrodes fabricated using varying CO₂ laser powers. (a) Optical images of 5 mm × 20 mm LIG electrodes fabricated with powers ranging from 2.5 W to 6.5 W.

To further evaluate the surface morphology, we acquired top-view scanning electron microscope (SEM) images of the surface of the six LIG electrodes fabricated using laser powers ranging from 2.5 W to 5.0 W. (**Figure S16**). The images reveal how LIG morphology evolves across the different power spectrum. While samples fabricated at similar power levels exhibit shared features, a clear overall trend can be observed. As laser power increases, the surface transitions from relatively uniform (2.5 W) to increasingly fragmented and porous (5.0 W), resulting in greater microstructuring and surface area. The 2.5 W sample shows controlled ablation, with granular features more visible at higher magnifications. Samples fabricated at 3.0 W and 3.5 W display more irregular and fragmented textures, due to more aggressive laser settings and more developed graphene structures. This trend continues in the 4.0 W to 5.0 W samples, with the 5.0 W surface exhibiting the highest degree of porosity and fragmentation. This morphological progression correlates with increasing laser power, resulting in LIG surfaces with enhanced electrical conductivity and larger surface area.


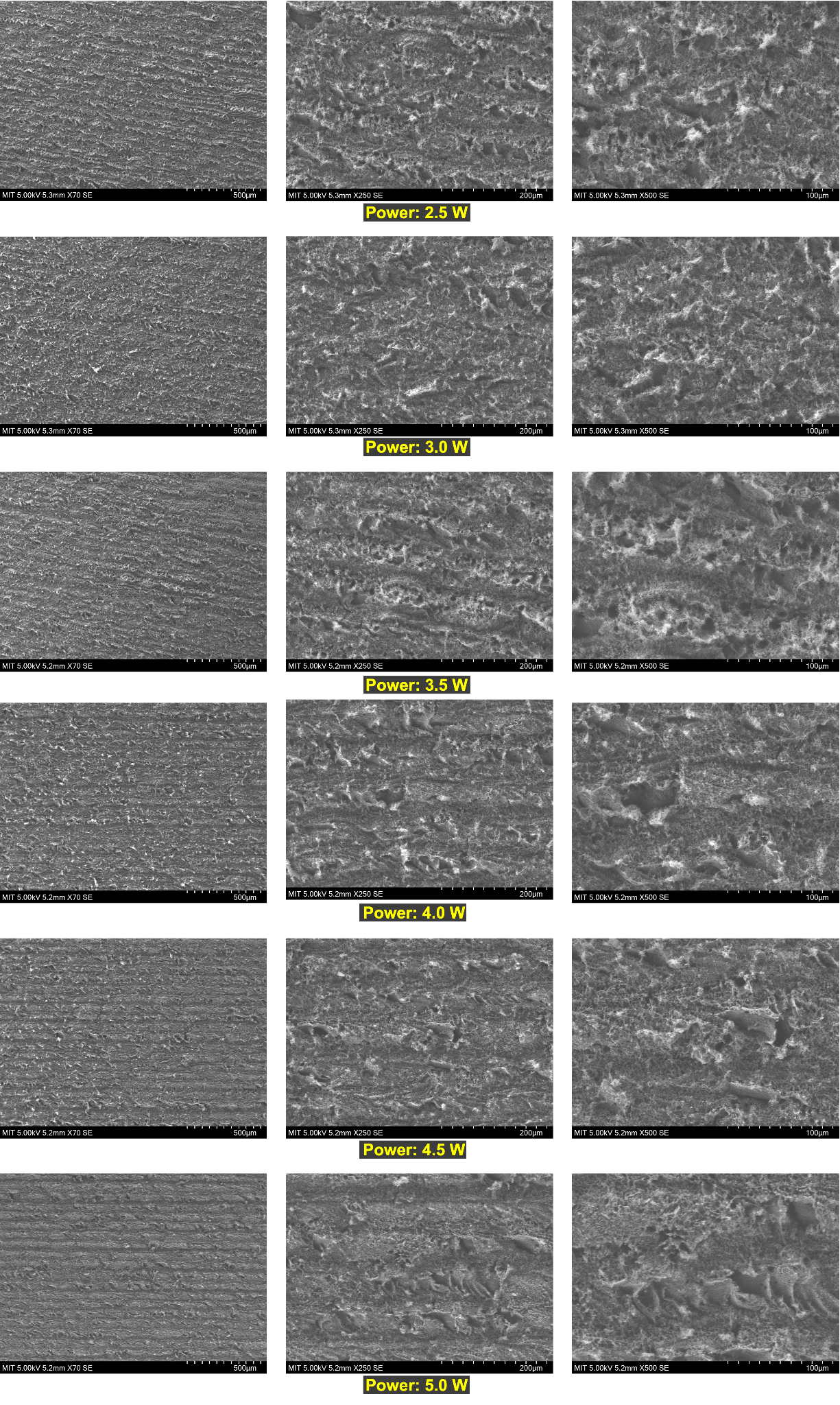


**Figure S16.** SEM images showing surface morphology of LIG formed at different laser powers.

We also quantified the electrical performance of these LIG electrodes by measuring their resistance. As shown in **Figure S17**, resistance decreases with increasing laser power, consistent with the expectation that higher laser power would induce more graphitization and improved electrical conductivity. This trend is in agreement with previous studies that report the correlation between laser power and LIG electrical conductivity or resistance ^[8–11]^ .


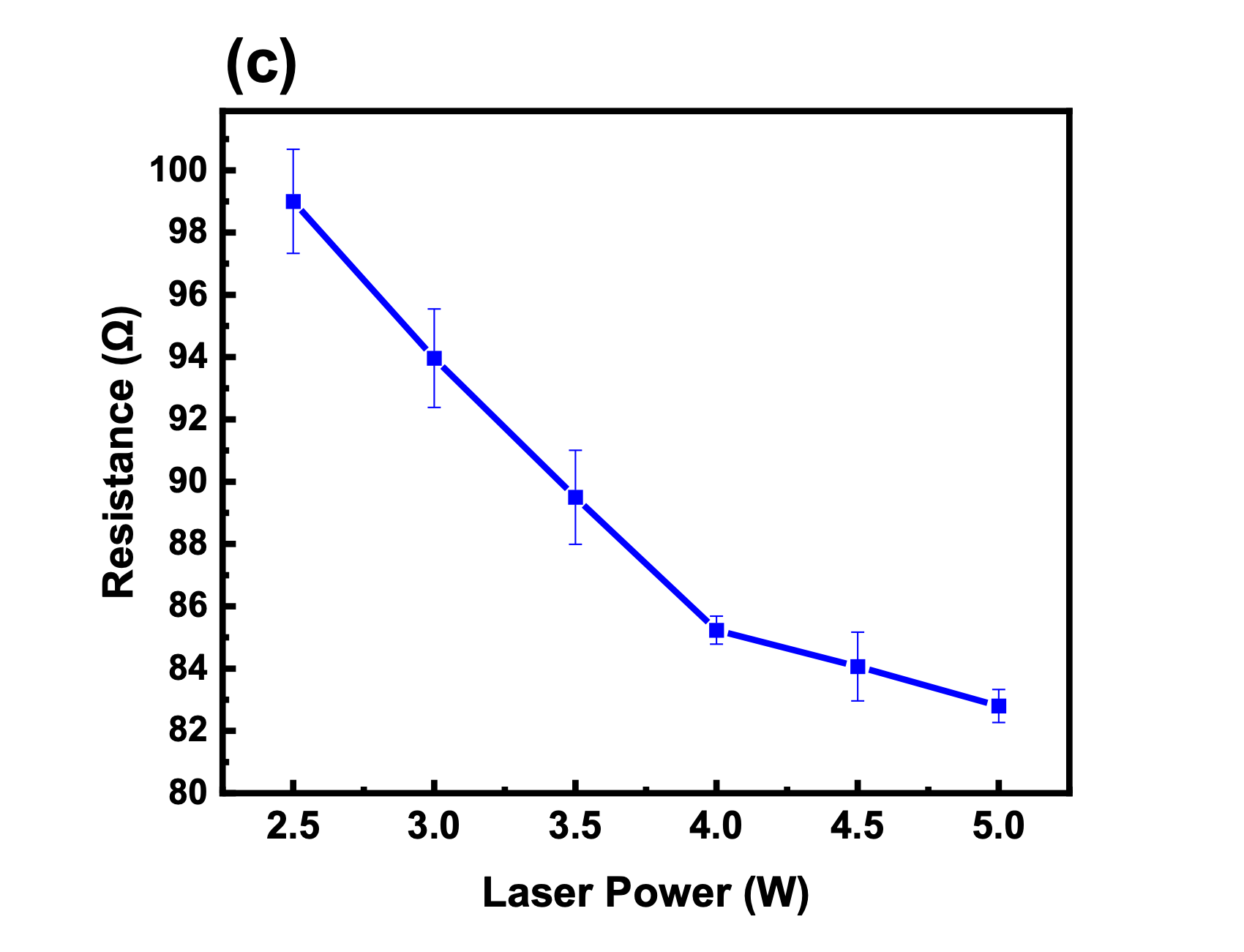


**Figure S17.** Resistance measurements of LIG electrodes as a function of laser power.

Finally, to study the effect of these material properties to device-level performance, we evaluated how LIG electrodes fabricated at different laser powers influence LMD movement. In these experiments, we used LIG both as a passive substrate and as an active cathode, as illustrated in F**igure S18a**, which includes schematic and example snapshots of the setup using 2.5 W LIG electrodes. To characterize the locomotion behavior, we applied two continuous electrowetting (CEW) voltages (9 V and 12 V) and tracked droplet displacement using automated tracking software. The extracted displacement profiles were used to calculate the average locomotion speed. As summarized in **Figure S18**, the LMD speed increases with increasing LIG laser power at both voltages, indicating a strong correlation between laser induced conductivity of the LIG electrode and its actuation efficiency. This trend is expected as LIG electrodes with higher conductivity enable more effective delivery of the applied potential, thereby generating stronger localized electric fields at the electrolyte interface. Detailed displacement profiles over time are shown in **Figure S18c** (9 V) and **Figure S18d** (12 V), showing an increase in locomotion speed as the laser power used to fabricate the LIG increases.

We selected 4.5 W laser power for the fabrication of LIG used in the experiments of the manuscript, as it provided an optimal balance between low electrical resistance (for efficient actuation) and strong adhesion to the polyimide substrate


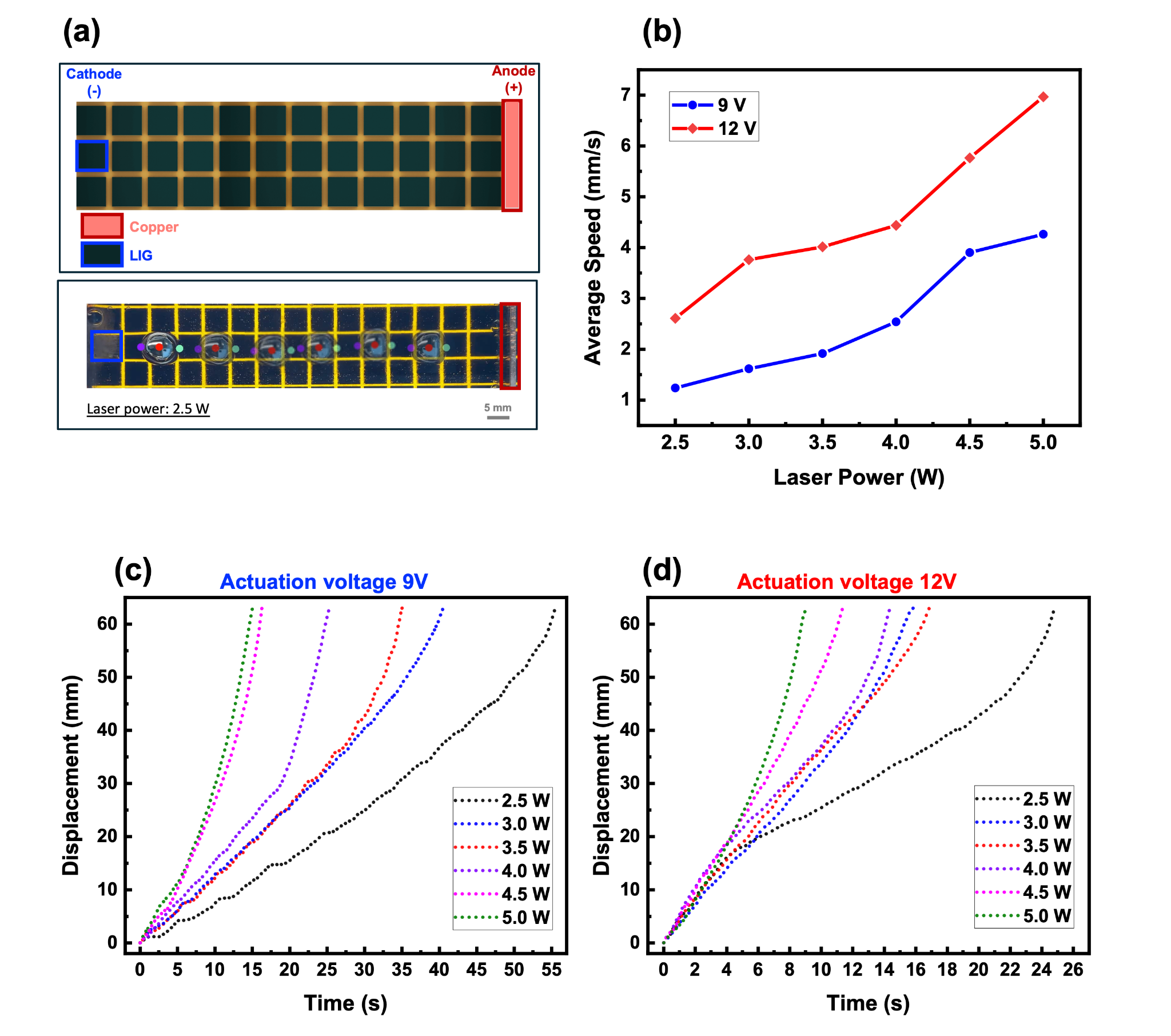


**Figure S18.** Liquid metal droplet (LMD) locomotion experiments using LIG electrodes fabricated at different laser powers. (a) Schematic and experimental snapshots of the test setup showing LIG used as an active cathode in a CEW configuration. (b) Average LMD speed as a function of laser power under 9 V and 12 V actuation voltages. (c,d) Displacement profiles of the LMD over time under 9 V (c) and 12 V (d) actuation voltages for LIG electrodes fabricated with powers ranging from 2.5 W to 5.0 W.

**Note S9: Key Parameters Influencing Liquid Metal Droplet (LMD) Locomotion Speed**

The locomotion speed of liquid metal droplets (LMDs) under CEW is governed by interplay various parameters. Broadly, these include the magnitude of the applied voltage, electric field distribution (electrode geometry, material type, and placement), electrolyte composition and concentration, droplet volume, substrate surface energy, temperature, and others. Many of these factors have been explored in the context of electrowetting or liquid metal actuation in previous literature ^[12,13]^. While a comprehensive parametric study is beyond the scope of this work, we have experimentally investigated three key factors that significantly impact LMD speed and are most relevant to our platform: (1) actuation voltage, (2) LMD volume, and (3) electrolyte concentration. The results are summarized in **Figure S19**.

**Figures S19a** and S19**b** show the displacement profiles and corresponding average speeds of droplets actuated using voltages ranging from 6 V to 12 V. Here, the LMD volume was fixed to 200 μL and the electrolyte concentration was 1M. As expected, increasing voltage results in higher speeds, due to the fact that stronger electric fields generate stronger interfacial tension gradients, which result in greater actuation forces and faster droplet movemen^[13–16]^. **Figures S19c** and **S19d** show the effect of droplet volume on speed. Larger droplets (300 μL) tend to move faster than smaller ones (100 μL). Here, the actuation voltage was fixed to 9V, and the electrolyte concentration to 1M. This result can be attributed to increased surface area at the droplet-electrolyte interface, which provides more interface for charge accumulation, Marangoni force generation and stronger overall driving forces ^[1,16]^. Additionally, larger droplets are better at overcoming resistance forces such as viscous drag and friction during locomotion

**Figures S19e** and **S19f** show that increasing NaOH concentration (0.1 M to 1 M) leads to higher actuation speed. Here the volume of the LMD was fixed to 300 μL and the voltage to 9V. Higher ionic concentration improves the conductivity of the electrolyte and increases the charge accumulation in the electrical double layer at the LMD-electrolyte interface, thereby inducing larger interfacial tension gradient. ^[14,15,17]^

For all experiments, we used an external pair of copper anode and cathode, a tracking algorithm to extract displacement profiles from the recorded videos and compute average speeds for consistent comparison.

Finally, we note that several system-specific factors also affect LMD speed in our setup. For example, we previously demonstrated that increasing the electrical conductivity of LIG electrodes (via increasing laser power) significantly improves actuation speed (Figure S18b). Additionally, the nature of the LIG pattern, whether a continuous film or grid pattern, also affects the movement speed, as shown in (Figures 2g and 2h of the main text). These findings further demonstrate the role of both general and system-specific factors in affecting LMD speed and dynamics.


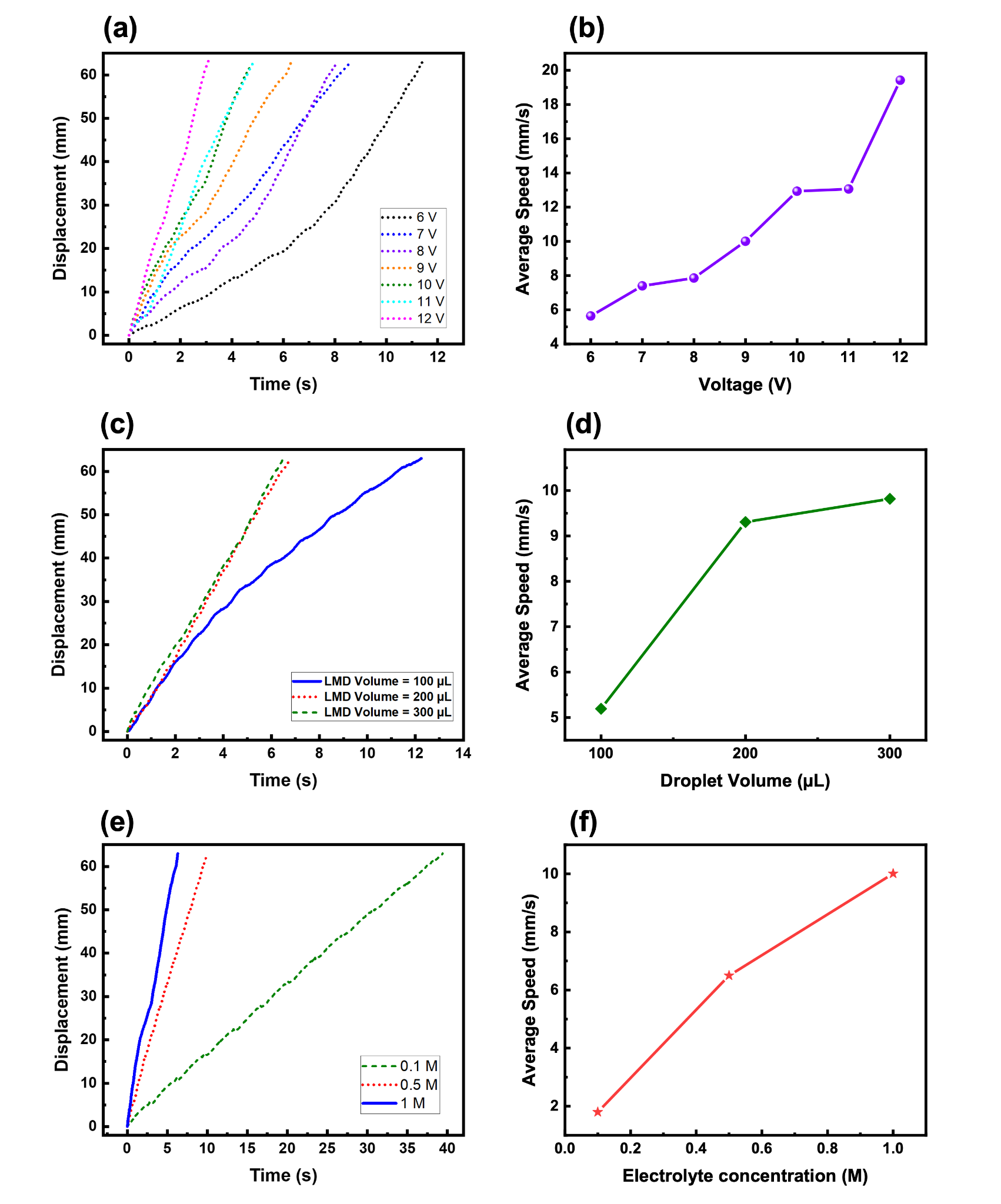


**Figure S19.** Effect of key parameters on the movement speed of liquid metal droplets (LMD) under CEW actuation. (a,b) Displacement profiles and average speed of LMD actuated at voltages ranging from 6 V to 12 V. (c,d) Displacement profiles and average speed for LMD of different volumes (100 µL, 200 µL, 300 µL). (e,f) Displacement profiles and average speed for LMD in NaOH electrolytes of varying concentrations (0.1 M, 0.5 M, 1 M).


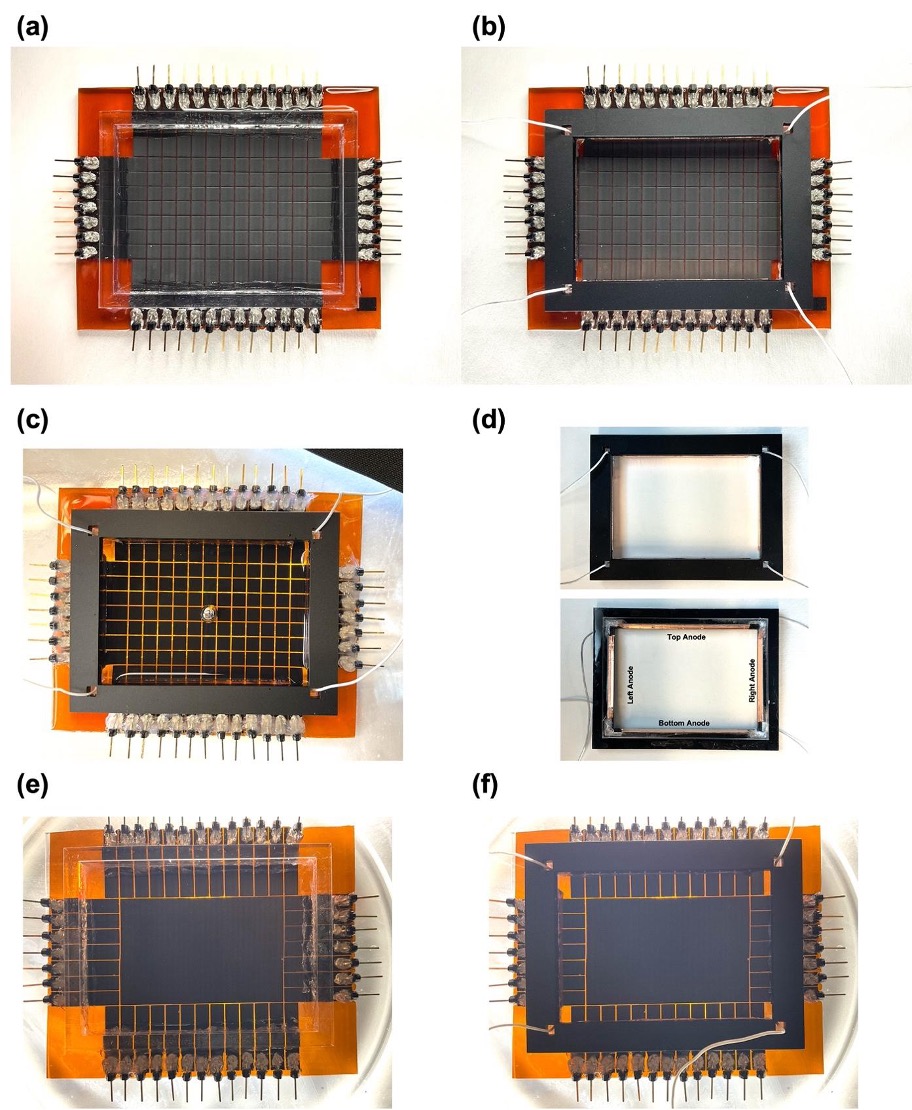


**Figure S20.** Assembly of the patterned LIG and LIG film platforms. (a-c) Assembly process for the grid-based LIG platform, showing the base platform with outer cathodes (a), the platform with the global anode frame added (b), and the platform filled with liquid metal (c). (d) Close-up of the global anode frame, highlighting the four individually addressable sides (top, bottom, left, right). (e, f) The assembly process for the LIG platform, showing the base platform with outer cathodes (e) and the platform after adding the global anode frame (f).

**Note S10: Influence of Grid Geometry and Droplet Volume on Liquid Metal Droplet Locomotion Behavior**

The geometric layout of the substrate plays a critical role in determining the ability of LMDs to retain a discrete shape and exhibit directional locomotion. We conducted a parametric study to systematically investigate how grid cell size (c), gap size (g), and droplet volume influence the discrete shape retention and directionality of locomoting LMDs.

As shown in **Figures S21-S24** and Table S3, we fabricated grid arrays with three different cell sizes (2.5 mm, 5 mm, and 7.5 mm) and three corresponding gap sizes for each (resulting in g/c ratios of 0.05, 0.1, and 0.4). These geometries were selected to explore the effects of spatial confinement and spacing on LMD locomotion.

All arrays were successfully fabricated using laser patterning, with the exception of the 2.5 mm grid with a 0.125 mm gap, which exceeded the resolution limit of the laser system and appeared under-resolved in the x- direction (as seen in **Figure S21**, top row).

Overall, the results show that both the grid geometry and droplet volume (size) play a critical role in determining whether the LMD maintains a discrete shape and directional movement over LIG. Across all tested conditions, LMDs consistently maintained a discrete droplet form, which means that the chosen gaps support droplet ability to remain discrete regardless of geometry. However, the directionality of locomotion, or the ability of the droplet to follow the intended field direction, was more sensitive to those geometric parameters. As previously mentioned in the main manuscript, LMD in electrolyte environment maintains its high surface tension which makes it prone to slipping across the surface in response to small disturbances. We found that gap sizes exceeding 0.375 mm frequently resulted in loss of directional control, likely due to LMD regaining its high surface tension and bouncy behavior allowing it to wander in the 2D platform. On the other hand, at intermediate g/c ratios of approximately 0.1, where the droplet size is comparable to the grid cell size, the LMD retained both discrete and directional behavior across all grid scales and volumes tested. At low g/c ratios (0.05) and a grid size of 5 mm with a 0.25 mm gap, the locomotion remained discrete and directional. However, for smaller grids ( 2.5 mm ) with similarly small g/c ratios, the droplet often lost directional stability, showing drift in its path. Overall, results suggest that g/c ~ 0.1 provides a regime for reliable discrete and directional locomotion of LMD and should be considered when designing the platform.


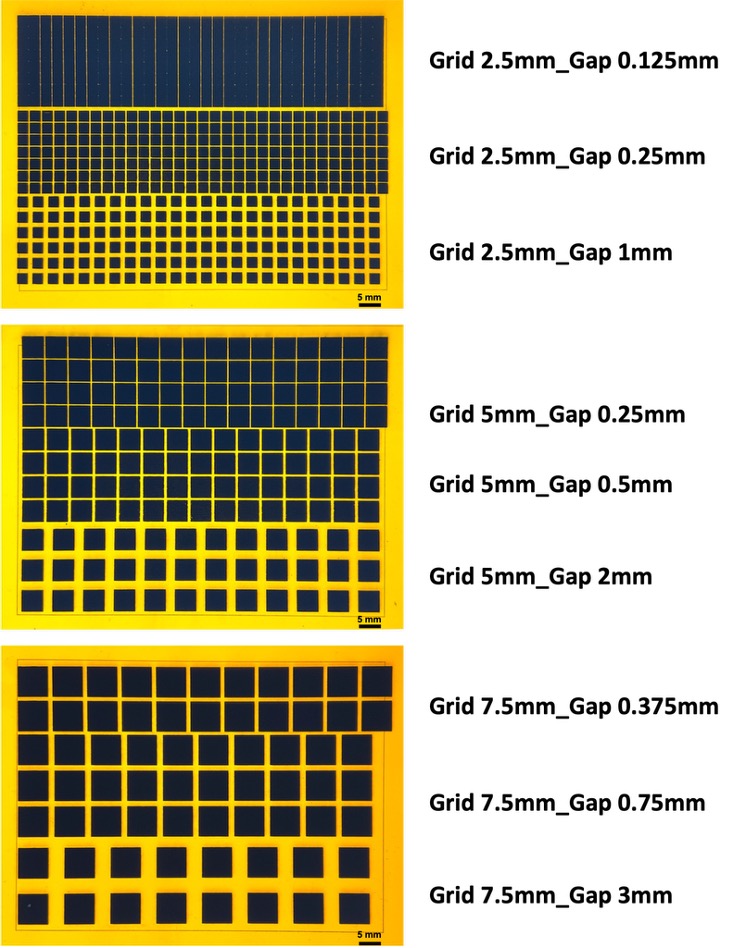


**Figure S21.** Fabricated grid arrays with varied grid cell sizes and inter-grid gaps used for evaluating liquid metal locomotion behavior. Each array consists of square unit cells (grid elements) arranged in a 2D lattice, with variation in both the cell size (c) and the gap size (g) between adjacent cells. The three arrays correspond to cell sizes of 2.5 mm, 5 mm, and 7.5 mm, each patterned with three gap conditions resulting in g/c ratios of 0.05, 0.1, and 0.4, respectively.


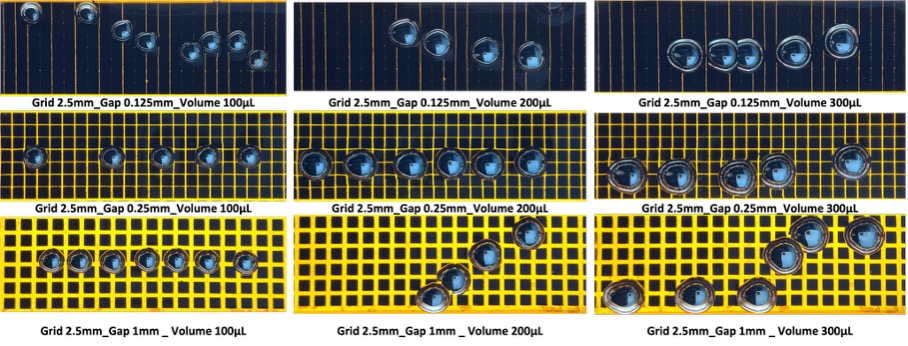


**Figure S22.** Sequential snapshots showing droplets with volumes of 100 µL, 200 µL, and 300 µL locomoting across grid arrays with 2.5 mm cell size and gap sizes of 0.125 mm, 0.25 mm, and 1mm.


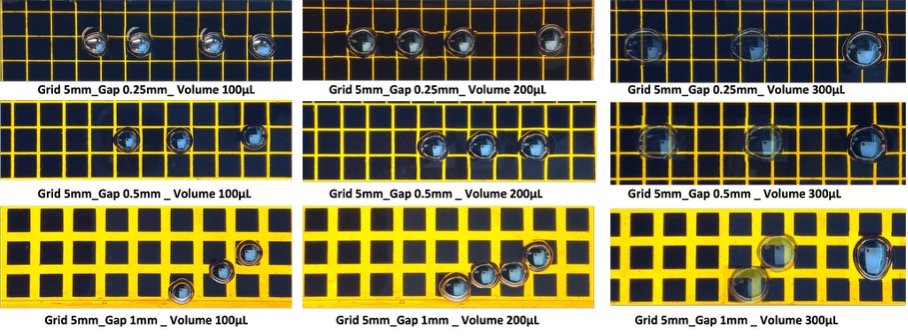


**Figure S23.** Sequential snapshots showing droplets with volumes of 100 µL, 200 µL, and 300 µL locomoting across grid arrays with 5 mm cell size and gap sizes of 0.25 mm, 0.5 mm, and 2 mm.


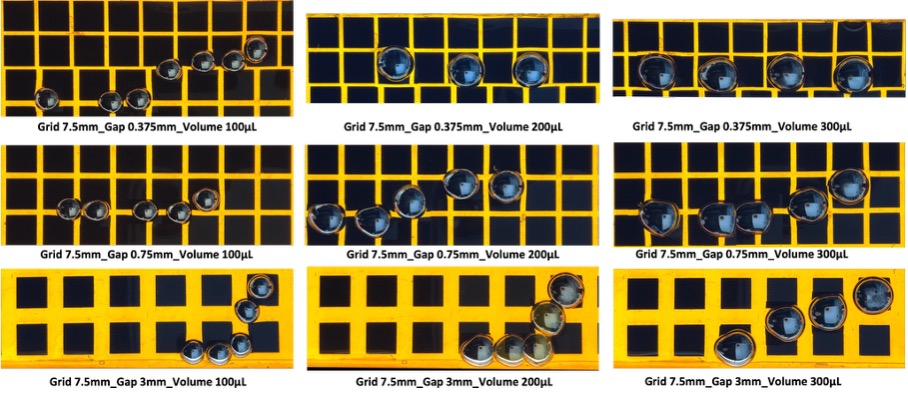


**Figure S24.** Sequential snapshots showing droplets with volumes of 100 µL, 200 µL, and 300 µL locomoting across grid arrays with 7.5 mm cell size and gap sizes of 0.375 mm, 0.75 mm, and 3 mm.

Table S3. Summary of liquid metal droplet locomotion behavior across various grid geometries and droplet volumes.

| **Grid Cell size c (mm)** | **Gap Size g (mm)** | **g/c Ratio** | **LM Behavior** | | |
| --- | --- | --- | --- | --- | --- |
| **LMD Volume** | | | **100 µL** | **200 µL** | **300 µL** |
| **2.5** | **0.125** | **0.05** | **Discrete, loss of direction** | **Discrete, loss of direction** | **Discrete, loss of direction** |
| **2.5** | **0.25** | **0.1** | **Discrete, directional** | **Discrete, directional** | **Discrete, partial loss of direction** |
| **2.5** | **1** | **0.4** | **Discrete + Directional** | **Discrete, loss of direction** | **Discrete, loss of direction** |
| **5** | **0.25** | **0.05** | **Discrete + Directional** | **Discrete + Directional** | **Discrete + Directional** |
| **5** | **0.5** | **0.1** | **Discrete + Directional** | **Discrete + Directional** | **Discrete + Directional** |
| **5** | **2** | **0.4** | **Discrete, loss of direction** | **Discrete, loss of direction** | **Discrete, loss of direction** |
| **7.5** | **0.375** | **0.05** | **Discrete, loss of direction** | **Discrete + Directional** | **Discrete + Directional** |
| **7.5** | **0.75** | **0.1** | **Discrete, loss of direction** | **Discrete, loss of direction** | **Discrete, loss of direction** |
| **7.5** | **3** | **0.4** | **Discrete, loss of direction** | **Discrete, loss of direction** | **Discrete, loss of direction** |

**Note S11: Electronics Integration for the 2D LIG Platform Control**

The experimental setup for controlling the 2D LIG platform is shown in **Figure S25**. It consists of a 2D platform, a custom control PCB, and an Arduino Mega, which handles the addressable switching of all electrodes. A DC power supply provides the CEW voltage. Additionally, a 5V power plug supplies power to key components on the control board. **Figures S25b** and **S25c** show the top and bottom views of the custom PCB, which was specifically designed to control the addressable electrodes, including global anodes and individually switchable cathodes. To enable programmable control over the 2D LIG platform, a custom Arduino Mega 2560 shield was designed to enable the selective actuation of the different electrodes. The control board shown in **Figures** **S26a** and **S26b** consists of power regulation components, relays, and transistor arrays to manage the four global anodes and the addressable cathodes. The board is powered by a 5V DC power input, which supplies the Arduino Mega as well as other control components. Additionally, terminal input allows an external DC power source, which is used to apply the CEW voltage. The switching architecture includes four addressable anode outputs, each controlled by relays, allowing for high-voltage switching. Additionally, 48 individually addressable cathode outputs are managed using NMOS transistors (MOSFETs), enabling programmable switching. This ensures independent control over each electrode, allowing dynamic voltage control across the platform.m The Arduino Mega 2560 acts as the central controller, coordinating the switching logic, which was controlled via the serial monitor. Figure S25c-d provides a detailed view of the circuit schematics, highlighting all the in the board. This hardware integration enables an electronically reconfigurable system, with selective and programmable voltage control across the 2D LIG platform. To enable easier control, a graphical user interface (GUI) was developed as a simulation tool (Figure S27) for adjusting valve potentials and CEW voltage in the valves and Y-shaped sorting channel. Future work will focus on extending it to the 2D platform and integrating it with the hardware.


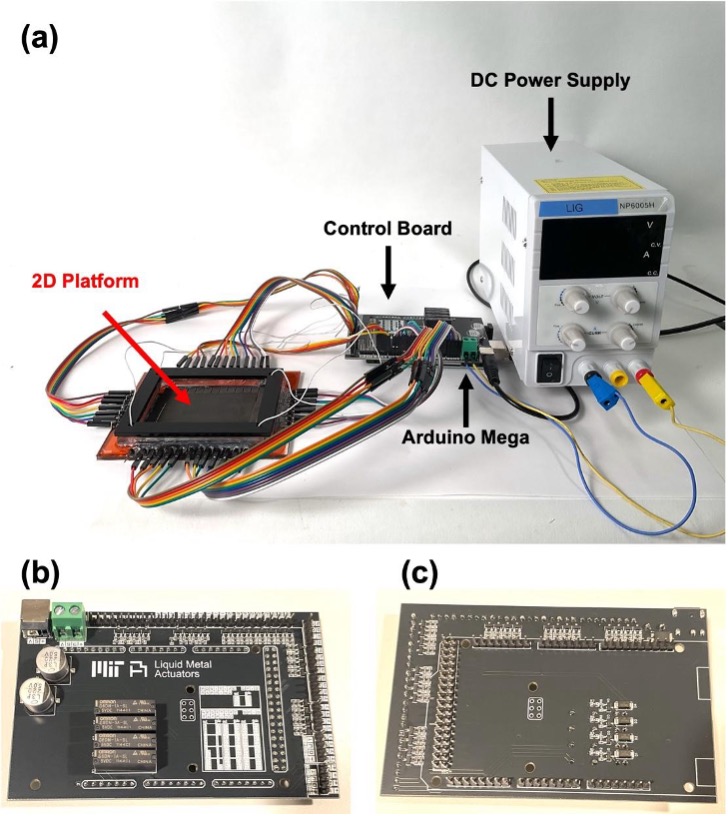


**Figure S25.** Electronics Integration. a) Experimental setup showing the 2D platform connected to the custom control board, Arduino Mega, and DC power supply. (b, c) Top and bottom views of the custom PCB designed to control the addressable electrodes, including the LIG cathodes and global anodes.


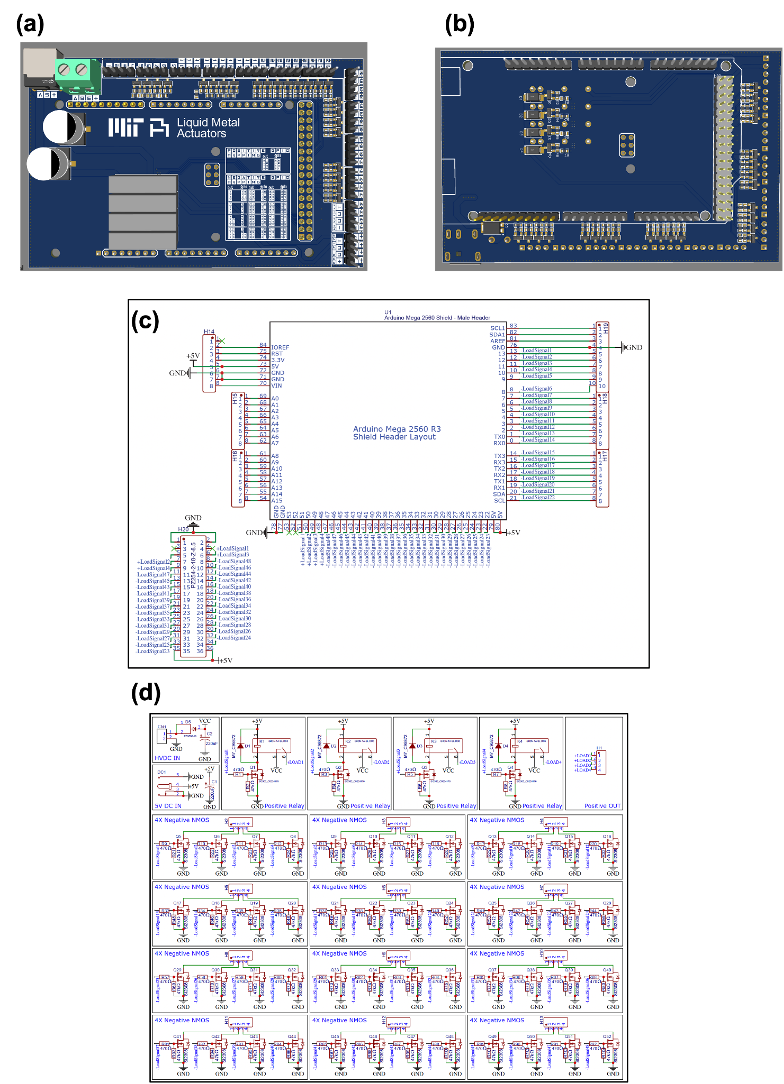


**Figure S26.** Custom control board design and schematics. (a, b) Top and bottom views of the custom PCB designed to interface with the 2D LIG platform. (c, d) Circuit schematics showing the component layout, including electrode, relay, and transistor arrays.


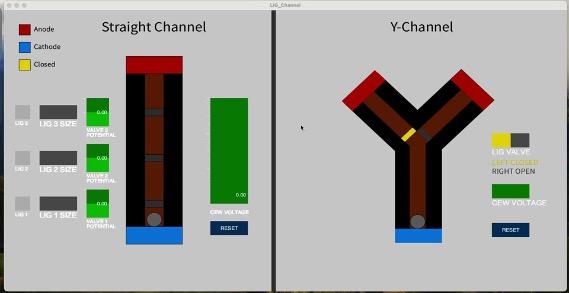


**Figure S27.** Custom graphical user interface (GUI) for LIG valve control. Snapshot of the custom-built GUI used to simulate and control the applied potential to each LIG valve within straight and Y-shaped channels. The interface allows independent adjustment of valve potential and continuous electrowetting voltage (CEW).


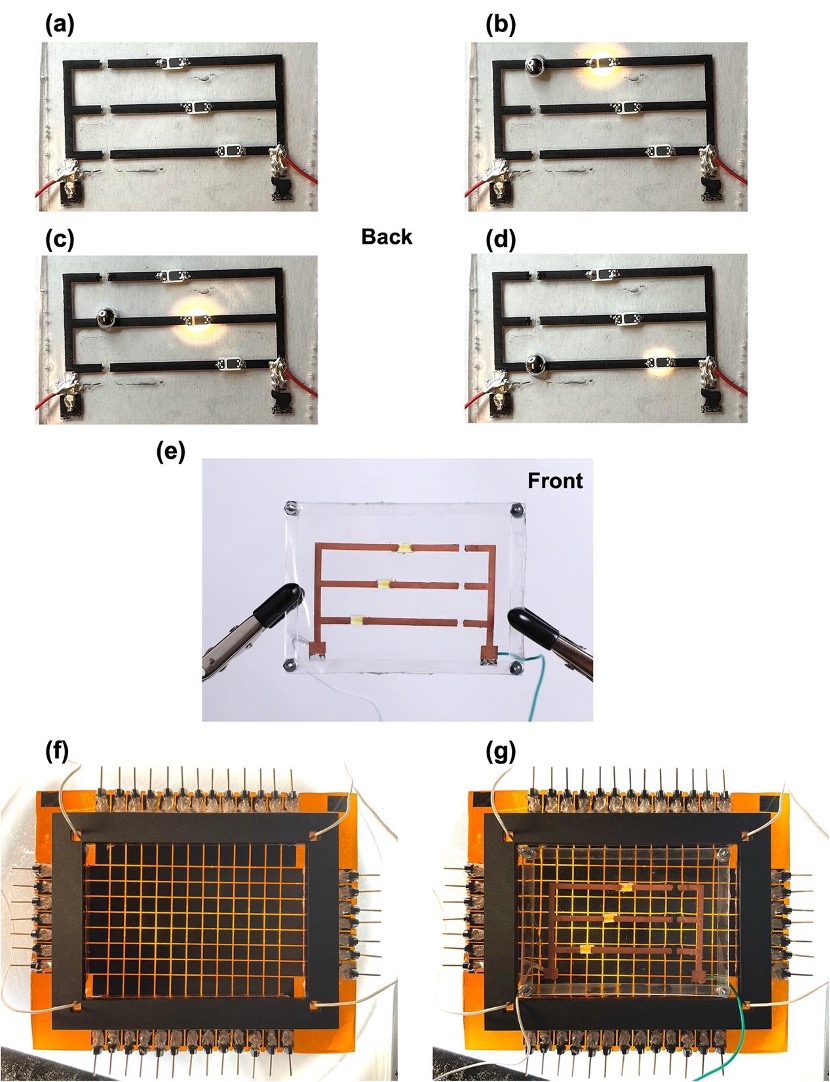


**Figure S28.** Layers of the reconfigurable circuit platform. (a-d) Back side of the copper-plated LIG circuit. LIG surface directly interfaces with LM, showing how the droplet functions as a movable interconnect to selectively activate LEDs. (e) the front side of the circuit, showing the highly conductive copper layer. (f-g) Fully assembled platform integrating both layers.


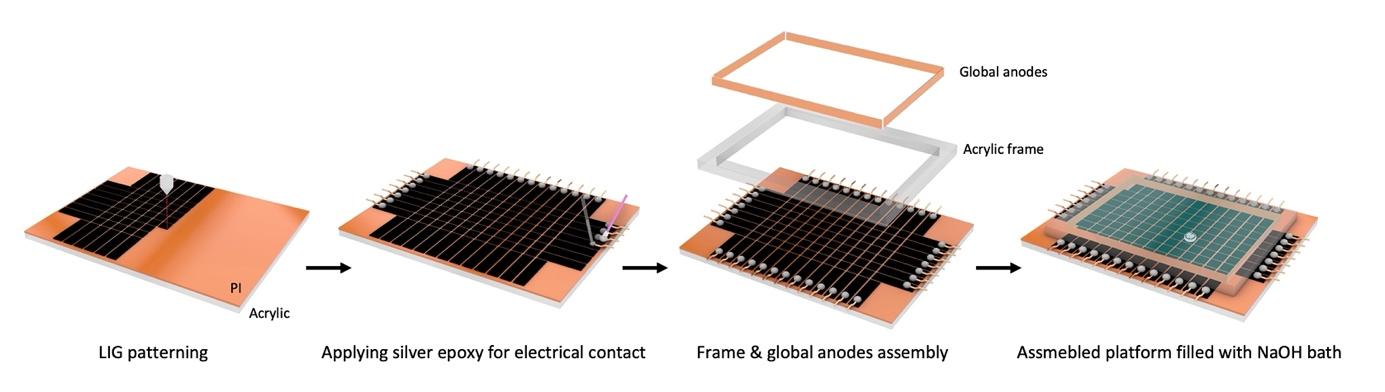


**Figure S29.** Fabrication process of the 2D LM platform. Step-by-step illustration of the platform assembly, beginning with laser patterning of LIG on a polyimide (PI) substrate, followed by establishing contacts with the cathodes using assembly, frame assembly, and adding LM and bath.

References

[1] C. B. Eaker, M. D. Dickey, *Appl. Phys. Rev.* **2016**, *3*, 031103.

[2] S.-Y. Tang, V. Sivan, K. Khoshmanesh, A. P. O’Mullane, X. Tang, B. Gol, N. Eshtiaghi, F. Lieder, P. Petersen, A. Mitchell, K. Kalantar-zadeh, *Nanoscale* **2013**, *5*, 5949.

[3] M. R. Khan, C. B. Eaker, E. F. Bowden, M. D. Dickey, *Proc. Natl. Acad. Sci.* **2014**, *111*, 14047.

[4] Y. Cui, Y. Ding, S. Xu, Z. Yang, P. Zhang, W. Rao, J. Liu, *Int. J. Thermophys.* **2018**, *39*, 113.

[5] E. B. Secor, A. B. Cook, C. E. Tabor, M. C. Hersam, *Adv. Electron. Mater.* **2018**, *4*, 1700483.

[6] R. C. Ordonez, C. K. Hayashi, C. M. Torres, N. Hafner, J. R. Adleman, N. M. Acosta, J. Melcher, N. M. Kamin, D. Garmire, *IEEE Trans. Electron Devices* **2016**, *63*, 4018.

[7] S. E. Fosdick, K. N. Knust, K. Scida, R. M. Crooks, *Angew. Chem. Int. Ed.* **2013**, *52*, 10438.

[8] M. Liu, J. Wu, H. Cheng, *Sci. China Technol. Sci.* **2022**, *65*, 41.

[9] C. Kincal, N. Solak, *Nanomaterials* **2024**, *14*, 879.

[10] R. G. Zonov, K. G. Mikheev, A. A. Chulkina, I. A. Zlobin, G. M. Mikheev, *Diam. Relat. Mater.* **2024**, *148*, 111409.

[11] Z. Wang, K. K. Tan, Y. C. Lam, *Micromachines* **2021**, *12*, 227.

[12] Junghoon Lee, Chang-Jin Kim, *J. Microelectromechanical Syst.* **2000**, *9*, 171.

[13] S.-Y. Tang, V. Sivan, K. Khoshmanesh, A. P. O’Mullane, X. Tang, B. Gol, N. Eshtiaghi, F. Lieder, P. Petersen, A. Mitchell, K. Kalantar-zadeh, *Nanoscale* **2013**, *5*, 5949.

[14] H. Ren, H. Jin, J. Shu, J. Xie, E. Wang, D.-A. Ge, S.-Y. Tang, X. Li, W. Li, S. Zhang, *Mater. Horiz.* **2021**, *8*, 3063.

[15] Q. Hu, T. Jiang, H. Jiang, *Materials* **2020**, *13*, 2122.

[16] S. Ghosh, R. Neupane, D. P. Sahu, J. Teng, Y. L. Kong, *Med-X* **2025**, *3*, 9.

[17] S. Bansal, Y. Tokuda, J. Peasley, S. Subramanian, *Langmuir* **2022**, *38*, 6996.

# Supporting Video Descriptions

**Video S1: Directional Switching of Liquid Metal Droplet Motion**

- **Description**: This video shows how applying an electrochemical potential directly to liquid metal reverses its motion under the effect of a continuous electrowetting (CEW) field.

**Video S2: Laser-Induced Graphene as a Compatible Interface for Liquid Metal Manipulation**

- **Description**: This video demonstrates the compatibility of laser-induced graphene (LIG) with liquid metal (LM). The first part compares LM deposition on LIG vs. copper, showing immediate alloying on copper. The second part shows LIG’s ability to reduce LM surface tension, making it easier to manipulate than on acrylic, where LM maintains its high surface tension. These properties make LIG a compatible interface for LM-based reconfigurable systems.

**Video S3: LMD CEW on Non-Graphitic vs Graphitic (LIG) Substrates**

- **Description**: This video demonstrates liquid metal droplet (LMD) locomotion under continuous electrowetting (CEW) on non-graphitic (PI) and graphitic (LIG) substrates. On the non-graphitic surface, the LMD moves toward the anode, following conventional CEW behavior. In contrast, on the graphitic (LIG) substrate, the LMD reverses direction and moves toward the cathode, showing the influence of surface properties on LM electrowetting dynamics.

**Video S4: LMD CEW on LIG Film vs Patterned- LIG Substrates**

- **Description**: This video compares liquid metal droplet (LMD) locomotion under continuous electrowetting (CEW) on LIG film vs grid-patterned LIG substrates. On the film surface, the LMD elongates and stretches due to continuous oxidation. In contrast, on the grid-patterned LIG, the LMD maintains its discrete droplet form, demonstrating how LIG substrate patterning influences LM's physical shape as it locomotes.

**Video S5: Operation of LIG LMD Valves**

- **Description**: This video demonstrates the functionality of LMD LIG-based ‘valves’, showing their normally open (NO) and normally closed (NC) states in both passive and active modes.

**Video S6: LMD Sorting in a Y-Shaped Channel Using LMD Valves**

- **Description**: This video demonstrates liquid metal droplet (LMD) sorting in a Y-shaped channel using programmable LIG-based ‘valves. By applying an oxidative potential to a valve at the inlet of a branch, the LMD is prevented from entering that branch and directed toward the open branch.

**Video S7: Buffer Gate Demonstration**

- **Description**: This video demonstrates a BUFFER gate using a normally closed (NC) LIG valve controlled by a reductive potential.

**Video S8: NOT Gate Demonstration**

- **Description**: This video demonstrates a NOT gate using a normally open (NO) LIG valve controlled by an oxidative potential

**Video S9: AND Gate Demonstration**

- **Description**: This video demonstrates an AND gate using two normally closed (NC) LIG valves arranged sequentially and controlled by reductive potentials.

**Video S10: OR Gate Demonstration**

- **Description**: This video demonstrates an OR gate using two normally closed (NC) LIG valves arranged side by side and controlled by reductive potentials.

**Video S11: NOR Gate Demonstration**

- **Description**: This video demonstrates a NOR gate using two normally open (NO) LIG valves, arranged sequentially, and controlled by oxidative potentials.

**Video S12: Linear Locomotion of LMD on a 2D LIG Platform**

- **Description**: This video demonstrates the controlled linear locomotion of a liquid metal droplet (LMD) on a 2D LIG platform. Outer cathodes are selectively activated with a corresponding global anode to guide the LMD, while the passive LIG grid in the center acts as a trapping site, stabilizing the droplet at specific locations.

**Video S13: Liquid Metal Manipulation on LIG Film: Elongation**

- **Description**: This video demonstrates liquid metal manipulation on a laser-induced graphene (LIG) film. Under applied electric fields, the LM elongates as it moves, showing controlled deformation. The video also shows directional motion along diagonal paths and LM-guided actuation, where the LM pushes a lever-like structure.

**Video S14: Reconfigurable Circuits**

- **Description**: This video shows liquid metal-enabled reconfigurable circuits. First, sequential LED activation is demonstrated as the LMD bridges paths one by one. Next, simultaneous activation is demonstrated as the LMD elongates to connect multiple LEDs. Finally, on-demand circuit repair restores a broken connection.

# 
